# Efficacy of Plasmid DNA Delivery into Mice by Intradermal Injections Alone and Facilitated by Sonoporation or Electroporation

**DOI:** 10.3390/vaccines14010082

**Published:** 2026-01-12

**Authors:** Daria Avdoshina, Vladimir Valuev-Elliston, Maria Belikova, Alla Zhitkevich, Anastasia Latanova, Galina Frolova, Oleg Latyshev, Ilya Gordeychuk, Ekaterina Bayurova

**Affiliations:** 1 Chumakov Federal Scientific Center for Research and Development of Immune-and-Biological Products of Russian Academy of Sciences, 119991 Moscow, Russia; mariabelikova60@yandex.ru (M.B.); zhitkevich_as@chumakovs.su (A.Z.); gluckiriapresent@yandex.ru (G.F.); gordeychuk_iv@chumakovs.su (I.G.); 2 Engelhardt Institute of Molecular Biology, Russian Academy of Sciences, 119991 Moscow, Russia; gansfaust@mail.ru (V.V.-E.); aalatanova@gmail.com (A.L.); 3 Gamaleya National Research Center for Epidemiology and Microbiology, 123098 Moscow, Russia; oleglat80@mail.ru; 4 Institute for Translational Medicine and Biotechnology, Sechenov University, 117418 Moscow, Russia

**Keywords:** DNA vaccine, mouse model, intradermal injection, ionic strength, electroporation, sonoporation, antibody response

## Abstract

**Background/Objectives**: A key disadvantage of DNA vaccines is ineffective uptake of plasmid DNA, resulting in low immunogenicity. A way to overcome it is forced DNA delivery, which requires specialized equipment and/or reagents. Effective delivery of plasmids without specialized devices or using commonly available ones would significantly increase DNA vaccine applicability. Here, we delivered DNA by intradermal injections, facilitating them by optimized sonoporation (SP) or electroporation (EP), and we compared these methods by their capacity to support the production of foreign proteins in mice. **Methods**: DNA delivery was optimized using the plasmid encoding firefly luciferase (Luc) (pVaxLuc). Luc production was assessed by bioluminescence imaging (BLI) (IVIS, PerkinElmer, Shelton, CT, USA; LumoTrace Fluo, Abisense, Dolgoprudny, Russia). Female BALB/c mice were injected intradermally (id) with pVaxLuc in phosphate buffers of varying ionic strengths. Injection sites were subjected to SP (Intelect Mobile, Chattanooga, UK) or EP (CUY21EDITII, BEX Co., Tokyo, Japan) or left untreated. Optimal delivery protocols were selected based on the highest in vivo levels of photon flux according to BLI. Optimal protocols for id injections with/without EP were applied to DNA-immunized mice with HIV-1 clade A reverse transcriptase. Antibody response induced by DNA immunization was assessed by ELISA. **Results**: The optimal phosphate buffers for id delivery had ionic strengths from 81 to 163 mmol/L. The optimal SP regimen included an acoustic pressure of 2.4 W/cm^2^ applied in a duty cycle of 2%. The optimal EP regimen included bipolar driving pulses of 100 V, a pulse duration of 10 ms, and an interval between the pulses of 20 ms. Optimized DNA delivery by id/SP injection was inferior to both id/EP and id alone. DNA immunization with HIV-1 RT by id injections induced anti-RT antibodies in a titer of 10^4^ and by id/EP in a titer of 10^5^. **Conclusions**: Electroporation of the sites of id DNA injection provided the highest levels of production of luciferase reporters and induced a strong antibody response against HIV-1 RT.

## 1. Introduction

DNA vaccines expanded in their applications from purely veterinary to stage III clinical trials against SARS-CoV-2 (INO-4800, AG0302-COVID19, and ZyCovD (https://covid19.trackvaccines.org/vaccines/#phase-3 accessed on 10 September 2025; [1]) and human papilloma viruses HPV16 and HPV18 causing cervical cancer (VGX-3100 DNA; [2]). DNA vaccines have multiple strong points. They use the host cell machinery to produce encoded protein antigens, reproducing the natural post-translation modifications of the immunogens [3]. They induce an effective cell-mediated and humoral immunity, mimicking that in the natural infection but circumventing the pathogen-induced immunomodulation [4,5]. They have an in-built adjuvant effect due to the presence of unmethylated CpG motifs characteristic of the plasmid DNA [6]. Last but not least, they are comparatively thermostable. Plasmid DNA only requires a cold chain for long-term storage [7]. Furthermore, new generations of thermostable DNA vaccines are on the way [8].

The main problem for the clinical use of DNA vaccines is low immunogenicity due to ineffective intracellular DNA uptake [7,9]. The uptake can be improved by forced DNA delivery using a wide variety of physical methods. The latter include microinjections, jet injections, hydrodynamic injections, ballistic transfection, sonoporation, optoporation, magnetoporation, and electroporation [10]. The majority of the above methods require specialized equipment, which limits their availability and increases the cost of vaccine application [11]. In addition, biosafety issues arise due to the risk of contamination of vaccination equipment, with potential transfer of contaminants from one vaccine recipient to another [12,13]. The necessity for physical protection and/or sterilization of the equipment components (such as electrodes or nozzles for biolistic delivery) and, eventually, personal protection, further increases the cost and complexity of DNA vaccination [14]. Effective methods for DNA vaccine delivery not requiring auxiliary equipment or using common appliances with no risk of contamination would significantly increase DNA vaccine applicability.

Classical methods of DNA vaccine delivery are needle injections, widely practiced in the early DNA vaccine applications and clinical trials ([15,16]; https://clinicaltrials.gov/study/NCT04591184, accessed 10 October 2025). The preferential route of DNA vaccine injection is intradermal (id) due to enhanced immune cell activation and effective lymph node targeting, hence superior immune response and also dose sparing [17,18].

One of the most potent methods to force DNA delivery by injection is EP. EP implies an electrophoretic transfer of the negatively charged DNA into the cells using an electric field, which causes reversible permeabilization of cell membranes. The efficacy of EP delivery is determined by the distribution of the electric field and its strength, defined by the type and geometry of the electrode, as well as the characteristics of the electrical pulses [19]. The method has proven itself to be reliable, efficient, and highly reproducible [20,21]. Unfortunately, no universal electroporation procedure exists due to the diversity of devices used to deliver electric pulses [20]. These drives continued optimization of the EP protocols.

An alternative, non-invasive method to force DNA delivery after the initial injection is sonoporation (SP). SP implies a temporal enhancement of the permeability of cellular membranes due to cavitation caused by the ultrasound and acoustic waves [22]. Cavitation is a nonthermal interaction between a propagating pressure wave and gaseous inclusions in a liquid (sub-micrometer gas bodies or cavities) [23]. Microscopic bubbles (“microbubbles”) exist naturally in the blood and tissues, serving as endogenous cavitation nuclei [23,24]. Low-pressure acoustic amplitudes cause their stable pulsation [23]. When the acoustic pressure amplitudes are increased, a violent collapse called inertial cavitation occurs, accompanied by an increase in the size of the natural microbubbles, followed by their explosion and emergence of shock waves and microstreaming, which altogether lead to the formation of temporary holes in the cell membrane [25]. By varying the parameters of ultrasound parameters, one can change the cavitation effects (size and behavior of microbubbles) and, consequently, the overall SP impact on the cell membrane. SP uses widely available miniature equipment for therapeutic ultrasound treatment [26]. DNA delivery by SP is well described and widely advocated [27,28,29,30]. However, the most promising results were demonstrated for SP used in combination with the commercially available (“exogenous” microbubbles) [https://articles.sonidel.com/] or self-produced microbubbles [30]. “Exogenous” microbubbles consist of a gas core encased by a stabilizing shell made from various biocompatible materials, lipids, proteins, biodegradable polymers, and surfactants. Specific materials are chosen to optimize properties like stability, size, and acoustic response [31]. “Exogenous” microbubbles enhance the cavitation [32,33] and increase SP efficacy, but at the same time, they reduce the availability of the method and increase delivery costs. The composition of the commercially available microbubbles brings up the issues of their histocompatibility and toxicity, as well as potential harm caused by the long-term accumulation in the body of their breakdown products [25]. While the pros and cons of “microbubble”-assisted SP are described in detail, an objective evaluation of SP effectiveness without the use of “exogenous” microbubbles, specifically compared to other DNA delivery methods, is missing.

The first step of DNA immunization is the injection of the solution of plasmid DNA. Buffers used to dissolve plasmid immunogens should be non-toxic and physiocompatible, should maintain DNA integrity, and should facilitate DNA uptake by the cells in vivo. The most common buffer is the phosphate-buffered saline (PBS); saline can also be used, although the latter has no buffering capacity, and both provide an isotonic environment [15]. Ionic strength strongly affects penetration of DNA into the cell [34]. Furthermore, in some types of forced DNA delivery, such as electroporation, the buffer should support conductivity and osmolarity. Altogether, this makes the ionic strength of the DNA solution an important basic parameter to optimize in both forced and non-forced delivery of plasmid DNA.

One should consider that plasmids are negatively charged, modulating the net ionic strength of the solution [35]. DNA penetration into the cell can be improved by increasing the ionic strength of the solution [34]; however, injection of DNA in the hypertonic solution would cause cell dehydration and shrinkage, potentially leading to cell detachment from the matrix, cell death, and tissue inflammation [34,36]. A hypotonic shock changes the cell membrane, allowing intracellular entry of large (up to 500 nm) nanoparticles [37]. Hypotonic shock causes cell death by swelling and rupture. However, mild hypotonicity (hypo-osmotic pressure) is a factor driving cell blebbing and symmetrical cell division and regulating protein folding, chromatin structure, transcription, and cell differentiation [38]. This makes mild hypotonic conditions attractive for DNA delivery; still, one has to define the threshold ionic strength of a buffer solution (minimum concentration of positively charged ions) at which the ionic balance in the injection mixture is not yet dramatically disturbed, does not prevent DNA from entering the cell, and does not interfere with cell viability and subsequent expression of the encoded protein.

Here, we have estimated the threshold of the ionic strength of the buffer at which DNA can still be effectively delivered by intradermal (id) needle injection and further compared the efficacy of plasmid delivery into mice after reinforcement of id delivery by sonoporation (id/SP) versus electroporation (id/EP). The efficacy of delivery was estimated by the levels of expression of a reporter protein. We have chosen firefly luciferase (Luc) as the activity of the Luc reporter in tissues, measured by bioluminescence, which directly correlates to the level of gene expression [39]. Id and id facilitated by SP or by EP were compared for the capacity to possibly express luminescent, and then the best methods were compared for the immune antibody response against the internal viral protein.

Intradermal DNA injections reinforced by SP were found to be the least efficient, being inferior to id injections reinforced by EP, and even to id injections alone. Superiority of DNA delivery by id/EP compared to id alone was illustrated by comparison of the latter two methods in their capacity to induce an immune response against an internal viral protein, HIV-1 reverse transcriptase (RT).

## 2. Materials and Methods

### 2.1. Plasmids

Plasmid encoding the firefly luciferase (pVaxLuc, [40]) was kindly donated by Dr Elizaveta Starodubova. Plasmid encoding the consensus reverse transcriptase (*RT*) of HIV-1 clade A FSU_A strain (pVaxRT-A) designed by us earlier [41] was subjected to site-directed mutagenesis, which introduced mutations D187N and D188N, abrogating the polymerase activity, and E480Q, abrogating RNase H activity, resulting in pVaxRT_Ain. Plasmids pVaxLuc and pVaxRT_Ain were produced in the *E. coli* Top10 strain (Invitrogen, Carlsbad, CA, USA) and purified using the EndoFree Plasmid Purification Mega Kit (Qiagen, Hilden, Germany). Purified plasmids were reprecipitated three times with 70% ethanol and dissolved in deionized water. Plasmid DNA concentration and purity were determined spectrophotometrically using a DS-11 spectrophotometer (DeNovix, Wilmington, DE, USA).

### 2.2. Animals and Animal Experiments

Experiments were carried out in compliance with the bioethical principles adopted by the European Convention for the Protection of Vertebrate Animals Used for Experimental and Other Scientific Purposes (Strasbourg, France, 1986) and the Order of the Ministry of Health of the Russian Federation dated 1 April 2016 No. 199n “On Approval of the Rules of Good Laboratory Practice”. Experimental procedures were approved by the ethics committee of the Gamaleya National Research Center for Epidemiology and Microbiology (permit No. 39 from 24 March 2023) and by the ethics committee of the Chumakov Federal Scientific Center for Research and Development of Immune-and-Biological Products of the Russian Academy of Sciences (protocol No. 170124-3 from 14 January 2024).

Female 8- to 12-week-old BALB/c mice (“Andreevka” or “Stolbovaya” breeding facility of the Federal State Budgetary Scientific Institution “National Center for Biomedical Technologies”, Moscow, Russia) were housed under a 12/12 h light/dark cycle with ad libitum access to water and food. Animals were acclimatized for two weeks before starting the experiments. 

The sample size for all series was calculated using the “resource equation” method [42].

Inhalation anesthesia in mice was induced using the 410AP Anesthesia Unit (Univentor, Zejtun, Malta) in a ventilated chamber containing air with 4% isoflurane and maintained with 2.5% isoflurane/air mix administered through a facial mask during intradermal injections, electroporation, and sonoporation. Upon completion of manipulation, mice were placed in a cage on a thermal insulation pad side by side to prevent hypothermia. At the experimental endpoint of the delivery optimization experiments, mice were euthanized by cervical dislocation. In immunization experiments, mice were euthanized by decapitation.

### 2.3. Injection of Plasmid DNA into Mice

DNA delivery protocols were optimized using plasmid encoding firefly luciferase (pVaxLuc). DNA immunization to induce an immune response was performed using a plasmid encoding inactivated consensus HIV-1 clade A reverse transcriptase (pVaxRT_Ain). Before injections of plasmid DNA, the animals were anaesthetized by inhalation, as described above, and hair was removed from the injection sites using an Aesculap Exacta trimmer (Aesculap, B. Braun Group, Melsungen, Germany). All DNA solutions were injected intradermally into the dorsal region at the base of the tail using an individual insulin syringe with a non-removable 29G needle at a dose of 50–100 µg per injection at a volume of 20 µL. The composition of the injected DNA solutions is listed in Table 1, Table 2, Table 3 and Table 4.

### 2.4. Optimization of Plasmid Delivery by Intradermal Injections

Experimental Series ID determined the threshold ionic strength of a buffer solution, allowing DNA delivery using solutions of plasmid DNA in phosphate buffers based on phosphate-buffered saline (PBS) (137 mM NaCl, 2.7 mM KCl, 10 mM Na_2_HPO_4_, and 1.8 mM KH_2_PO_4_) of decreasing ionic strength. Groups of mice (n = 5) received injections in two sites, one to the left and one to the right from the base of the tail, of 100 µg of pVaxLuc dissolved in serially diluted phosphate buffers. Solutions were prepared by adding the calculated volume of 10x PBS to the plasmid stock and then making up the injection mixture volume to 20 µL with deionized water (H_2_O) (Table 1). The resulting ionic strength of the solutions, calculated as described (https://byjus.com/chemistry/ionic-strength-unit/, accessed on 8 June 2023), is presented in Table 1.

### 2.5. Optimization In Vivo Delivery of Plasmid DNA by Sonoporation

Experimental Series SP determined the optimal conditions of plasmid delivery by the id/SP by varying the duration and intensity of acoustic pulses (Table 2). Groups of mice (n = 3) received id injections of 50 µg pVaxLuc in phosphate buffer with an ionic strength of 163 mmol/L (isotonic phosphate buffer) in one site to the left of the base of the tail. After id DNA injection, the injection site was treated with acoustic gel, the sensor head of the ultrasound generator sensor was applied, and sonoporation was performed using a portable ultrasound generator using a 2 cm^2^ sensor (both Chattanooga, Guildford, UK) in the pulse mode, with the transducer operating at a frequency of 1 MHz. The range of acoustic intensities used was determined based on the ultrasound generator and tissue parameters using Formula (1) [43] as follows:

(1) $$I=\frac{p^{2}}{2\rho v}$$

where *p* is the acoustic pressure value, Pa; *ρ* is the tissue density, kg/m^3^; and *v* is the propagation velocity of ultrasonic waves in tissue, m/s. The boundary conditions for SP were determined according to [44,45]. The minimum acoustic intensity was 0.6 W/cm^2^, and the maximum intensity was about 3.7 W/cm^2^, according to calculations using Formula (1). The average propagation velocity of ultrasonic waves in soft tissues was assumed to be 1540 m/s [46], and the density of the dermis was assumed to be up to 1200 kg/m^3^ [47]. Increasing working acoustic intensities of 0.6, 1.2, and 2.4 W/cm^2^ were selected, with the latter approaching the maximum intensity of the ultrasound generator in use (3 W/cm^2^). The duty cycle values were selected based on the length of the time that an ultrasound wave was being transmitted (pulse duration), 1 or 2 ms, and a 16 Hz pulse repetition frequency was used (PRF) (2% and 3%, respectively, https://binaryupdates.com/pwm-in-lpc2148-arm7/equation-for-calculation-duty-cycle/, accessed 10 September 2022). Prolonged sonication has been reported to lead to cell lysis [30]. Therefore, the total sonication time for the DNA injection areas was set to a maximum of 3 s.

### 2.6. Optimization of In Vivo Delivery of Plasmid DNA by Electroporation

Experimental Series EP-1 determined the optimal conditions of plasmid delivery by id/EP, including amplitude (voltage), polarity, pulse duration, and frequency of the driving pulses of the electric field applied by an electroporator (Table 3). Groups of mice (n = 4) received single id injections of 50 µg of pVaxLuc in isotonic phosphate buffer (163 mmol/L) at one site to the left of the base of the tail. Thereafter, injection sites were electroporated using a CUY21EDITII device (BEX Co., Ltd., Tokyo, Japan) with a multi-needle electrode (1.5 × 4 mm; Cellectis, Paris, France) operating in two stages: one poration pulse with constant parameters and then a series of eight driving pulses of variable parameters (Table 3). The poration pulse parameters were as follows: a voltage of 400V and a pulse duration of 50 ms, with an interval of 50 ms between the poration pulse and the subsequent driving pulses. The duration of the pulses was chosen to be shorter than the plasma membrane charging time constant (from µs to ms) to ensure changes to the plasma membrane [48]. Bioluminescence signal significant Luc gene delivery and expression was registered using IVIS (Perkin Elmer, Shelton, CT, USA) (see Section 2.8 for details).

In Series EP-2, mice were electroporated using a similar scheme to group EP-1.5 (Table 3), but mice received id injections of 100 µg of pVaxLuc with isotonic phosphate buffer (163 mmol/L) in two sites, one to the left and one to the right from the base of the tail (Table 1). Bioluminescence signal was detected using LumoTrace Fluo (Abisense, Dolgoprudny, Russia) (see Section 2.8 for details).

### 2.7. DNA Immunization with HIV-1 RT

Experimental Series IR determined the capacity of id injection alone and facilitated by EP to induce an immune response by the delivery of plasmid encoding HIV-1 RT (pVacRT_Ain). Groups of mice (n = 5) received prime and boost id injections of 100 µg of pVaxRT_Ain in phosphate buffers with ionic strengths of 163, 81, and 0 mmol/L (deionized H_2_O). One group (IR.4 *; Table 4) received prime and boost id injections of 100 μg of pVaxRT_Ain in isotonic phosphate buffer (163 mmol/L), followed by EP using the optimal regimen defined in Series EP-1 (EP-1.5 group; Table 3) (see Section 2.6). A group of naïve mice served as a negative control (IR.5; Table 4).

### 2.8. In Vivo Bioluminescent Imaging (BLI)

The efficiency of plasmid delivery and subsequent Luc reporter expression in mice was assessed by bioluminescence imaging (BLI) of the sites of pVaxLuc injections presented as the regions of interest (ROIs). BLI was performed on the following days after plasmid delivery: 2 to 3, 5 (all methods), 9, and 15 (id/SP, id/EP), and for id/EP, it was also performed overnight/16 h after DNA delivery (day 1). Before monitoring, mice were injected intraperitoneally with freshly prepared D-luciferin (PerkinElmer) at a dose of 150 ug per g of mouse weight. After a 10 min incubation period, allowing the substrate to be distributed uniformly throughout the body, mice were anaesthetized with a mixture of isoflurane with oxygen initiated at 4% and monitored by BLI while being maintined under anesthesia with 2.5% isoflurane oxygen mixture.

In Series EP-1, Series SP bioluminescence monitoring was performed using IVIS (PerkinElmer), and in Series ID and EP-2, by LumoTrace^®^ Fluo (Abisense). Both the Perkin Elmer and Abisense devices are equipped with CCD image sensors, which convert photons into electrical charges, and 16-bit analog-to-digital converters (ADCs) are used to convert the analog signal from the CCD to a digital signal (named ADC counts, or simply “counts”). The digital output signal (“counts”) from each pixel is proportional to the number of photons on it from the light source. The technical specifications of the IVIS CCD image sensor can be found in the IVIS Spectrum Hardware Manual at https://www.manualslib.com/manual/1649648/Perkinelmer-Caliper-Ivis-Spectrum.html. (accessed 3 February 2023). LumoTrace^®^ Fluo was equipped with a CCD Grade 1 2688 × 2200 image sensor, with an operation range of 400 to 1000 nm, a field of view (FOV) of 25 × 25 cm, and a registering pixel size of 4.54 × 4.54 µm.

BLI data obtained using IVIS was processed using Living Image 4.5 Software^®^ (Perkin Elmer). Living Image 4.5 Software converts the digital “counts” within the ROI into a value at each pixel, summed or integrated over the ROI area or total flux (photons/sec) (IVIS Spectrum Software Manual, https://www.biotech.cornell.edu/media/235, accessed 3 February 2023). BLI data obtained by LumoTrace was processed using Icy software (BioImage Analysis Lab at Institut Pasteur, licensed under GPLv3; Institut Pasteur and France-BioImaging, https://icy.bioimageanalysis.org/). Icy software presents output data in the form of ADC counts (without mathematical processing). With this, the signal values measured from the ROI in images obtained by the LumoTrace device represent the values of all digital signals from each pixel, summed or integrated over the ROI area, or “total counts”. This data was presented as total flux (IVIS) or total counts (LumoTrace) and also as % of the maximal values observed on day 2 by each of the methods, which allowed us to compare the results. In a head-to-head comparison, the efficacy of the delivery of pVaxLuc by different methods on day 2 was presented as a % of the efficacy of the delivery of pVaxLuc by id injection in isotonic phosphate buffer (163 mmol/L), followed by optimal EP assessed on the same imager and taken as 100%.

### 2.9. Assessment of Anti-RT Antibody Response by ELISA

Recombinant HIV-1 RT-A protein (rRT-A) was expressed in *E. coli* as a 6-His-tagged protein and purified by Ni-agarose column chromatography, as described by us previously [41]. Mouse sera were pre-blocked by incubation with PBS containing 10% normal goat serum, 2% bovine serum albumin, and 0.05% Tween 20 (Scan Buffer) overnight at 9 °C. The 96-well flat-bottom ELISA plates (MaxiSorb, Nunc A/C, Roskilde, Denmark) were coated with rRT-A dissolved in PBS at a concentration of 0.3 ug/mL and incubated at 9 °C overnight. Plates were washed six times with PBS containing 0.05% Tween 20 and dried over a paper towel. Thereafter, mouse sera were loaded into the wells with six serial four-step dilutions starting from 1:200 and incubated overnight at 9 °C. After incubation, plates were washed five times with PBS containing 0.05% Tween 20 and incubated for 1.5 h at 37 °C with goat anti-mouse IgG conjugated to HRP (ab6789, Sigma, Burlington, MA, USA) diluted in a Scan Buffer. Thereafter, plates were washed as described above, dried by repeated tapping on a paper towel, and incubated at room temperature with 100 µL of freshly made 3, 3′, 5, 5′-Tetramethylbenzidine (TMB) substrate in the dark until the appearance of colorimetric reaction (normally 10 min). The reaction was stopped by the addition of 50 µL of 0.1 M sulfuric acid. Optical density was measured at a dual wavelength of 450 and 620 nm using a spectrophotometer (Multiskan, Thermo Fisher, Waltham, MA, USA). Optical density values were represented as OD_450_–OD_620_ (OD_450–620_). The endpoint dilution titer was established as the serum dilution at which the OD_450–620_ of the hyperimmune mouse serum became equal to or lower than the average OD_450–620_ of 3 to 5 control mouse sera assessed on the same plate + 3SD or an OD value of ≤0.1; the higher of the values was selected. The final antibody titers were calculated from titration curves approximated by a nonlinear regression model—the 4PL curve—as recommended (https://www.biolegend.com/en-gb/blog/curve-fitting-for-immunoassays-legendplex, accessed 30 April 2024).

### 2.10. Statistical Analysis

Statistical analysis of the data was performed using GraphPad Prism 8.0.1 (Prism, San Diego, CA, USA). Group comparisons were performed using the Kruskal–Wallis test, and pairwise comparisons were performed using the Mann–Whitney test. Comparisons in experimental groups were performed using one-way analysis of variance with Dunnett’s multiple comparison correction. Values of *p* < 0.05 were considered significant.

## 3. Results

### 3.1. Effective Expression of DNA After Intradermal Injection Requires an Isotonic Buffer

**Figure 1:**
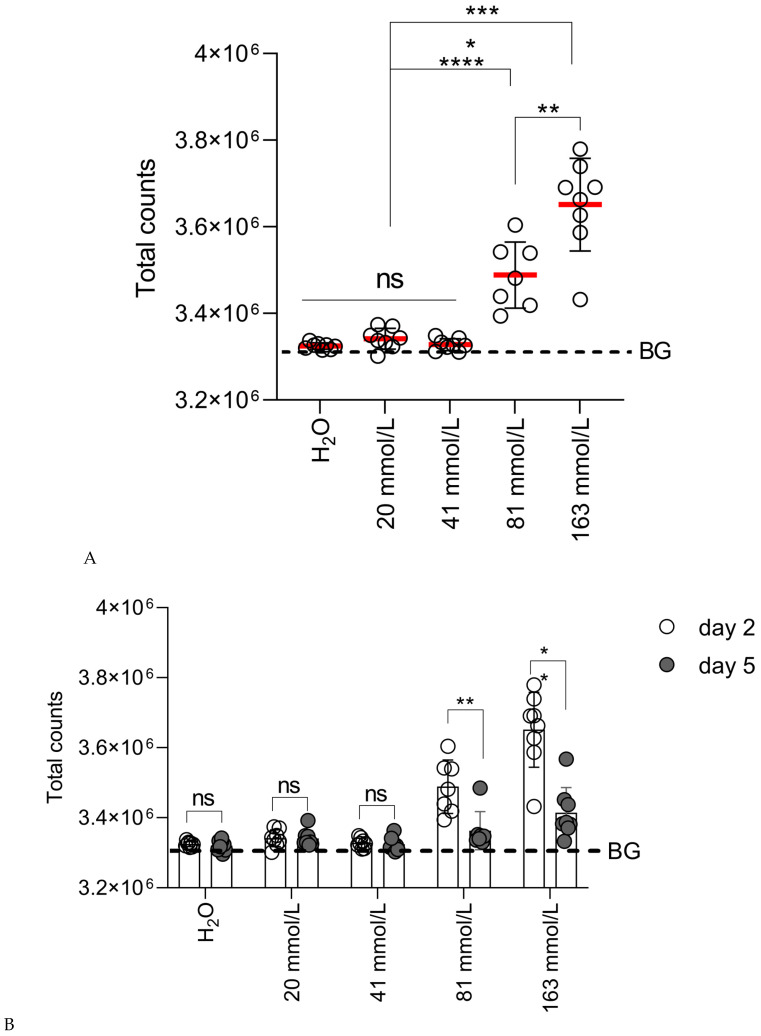

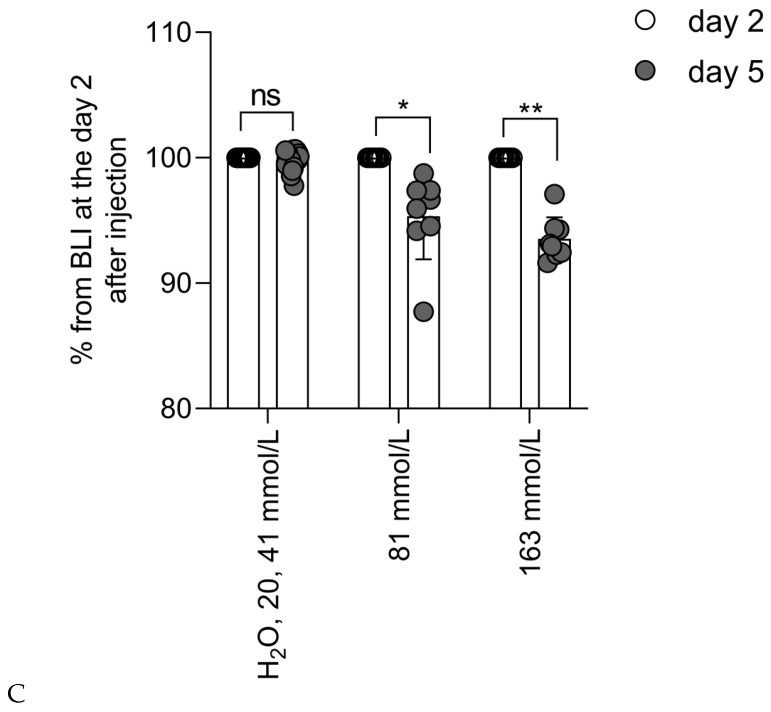
Levels of bioluminescence from the sites of id injection of pVaxLuc delivered in phosphate buffers with ionic strengths of 20, 41, 81, and 163 mmol/L or deionized H_2_O (Series ID; Table 1). (**A**) Comparison of photon emission in total counts (see Section 2.8) from the sites of plasmid injections in buffers of given ionic strengths on day 2. (**B**) Comparison of the decrease in photon emission (total counts) from day 2 to day 5 in mice receiving pVaxLuc in phosphate buffers of given ionic strengths. (**C**) Comparison of the decrease in photon emission from day 2 to day 5 (in %) in mice receiving pVaxLuc in phosphate buffers with ionic strengths of 163 mmol/L and 81 mmol/L and of 41 mmol/L, 20 mmol/L, and deionized H_2_O taken together (as the latter three groups did not differ, *p* > 0.1). The background level of bioluminescence (BG) (dashed line) was set as the average signal from the area of the body equal to the ROI of naïve BALB/c mice (n = 3) injected with D-luciferin + SD. The background counts were attributed earlier to autoluminescence [53]. All data are presented as the mean value within each group ± SD. Pairwise comparisons are performed using the non-parametric Mann–Whitney test; *p* ****—*p* < 0.0001; *p* ***—*p* < 0.001 *p* **—*p* < 0.01; *p* *—*p* < 0.05; ns—not significant.

The first step was to optimize the initial delivery of plasmid DNA. For this, BALB/c mice were intradermally (id) injected with plasmid DNA encoding the firefly luciferase gene (pVaxLuc) in phosphate buffers of varying ionic strengths (from 81 to 0 mmol/L) and were compared to the control group, which received pVaxLuc in a phosphate buffer with an ionic strength of 163 mmol/L (isotonic phosphate buffer) (Series ID; Table 1). Monitoring of luciferase expression by BLI was performed on days 2, 3, and 5 post-injection. In all groups, the maximal level of Luc expression was reached on day 2, concordant with the earlier studies (see, for example, [49,50]). The highest level of photon emission (Luc expression) was reached when DNA was delivered in an isotonic phosphate buffer (163 mmol/L), followed by a buffer with an ionic strength of 81 mmol/L (Figure 1A). The bioluminescence signals from the injection sites in the other three groups (41, 20, and 0 mmol/L (deionized H_2_O)) were at the level of autoluminescence of naïve mice (Figure 1A). Thus, DNA delivery in solutions with an ionic strength below 81 mmol/L was ineffective, providing negligible levels of reporter expression with no difference between the groups.

The immunogenicity of firefly luciferase is comparatively low. In mice, this allows long-term (up- o two months) monitoring of Luc expression after Luc DNA injection [39]. Still, when overexpressed, Luc induces cellular response, causing a gradual decrease in BLI signal and reflecting a decrease in Luc expression due to immune-mediated loss (killing) of the expressing cells [40,51]. The priming phase of immune response peaks at 48–72 h post-exposure [52]. Based on this, a decrease in photon emission after day 2 was suggested earlier as a surrogate marker of the development of anti-Luc immune response [40]. Here, we observed a decrease in the bioluminescence signal starting from day 5 in mice receiving pVaxLuc in phosphate buffers with ionic strengths of 81 and 163 mmol/L, whereas a change was observed in mice receiving pVaxLuc in buffers with ionic strengths of ≤41 mmol/L (Figure 1B,C).

Thus, all DNA deliveries in phosphate buffers performed at the ionic strength of 41 mmol/L and below were equally ineffective. The ionic strength of the phosphate buffer of 81 mmol/L was a threshold beyond which gene expression after id DNA injection was still supported. The highest level of reporter expression after id injection was achieved in a phosphate buffer with the physiological ionic strength.

### 3.2. Optimization of Forced DNA Delivery by Sonoporation

The next step was to test if DNA delivery after initial id injection can be facilitated by sonoporation (id/SP) (Series SP; Table 2). In the SP series, BALB/c mice received id injection with pVaxLuc in an isotonic phosphate buffer (163 mmol/L), defined as optimal in the ID series. Injection sites were subjected to sonication in varying conditions (Table 2). Firstly, we assessed the effect on sonication delivery of the changes in the acoustic pressure; other parameters (pulse repetition rate, pulse duration, and exposure time) were kept unchanged. Three acoustic pressure options in the range of 1 to 2.1 kPa were selected, corresponding to the acoustic intensities of 0.6, 1.2, and 2.4 W/cm^2^ with a pulse duration of 1 ms. To minimize the risk of cell lysis, we selected a duty cycle of 2%, corresponding to a series of acoustic oscillations lasting 1 ms [30]. Photon flux from the injection sites reflecting Luc reporter expression was set to be monitored at days 2, 3, 9, and 15, as we expected initially high levels of Luc expression with a gradual increase.

**Figure 2:**
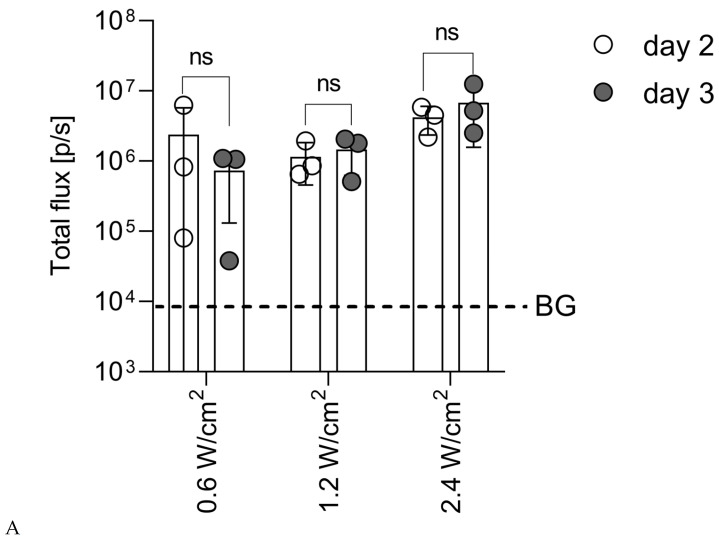

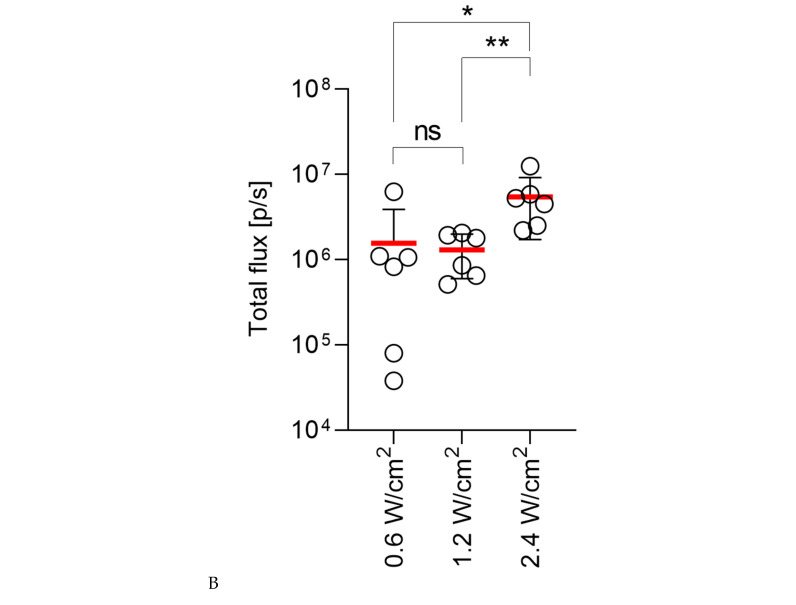

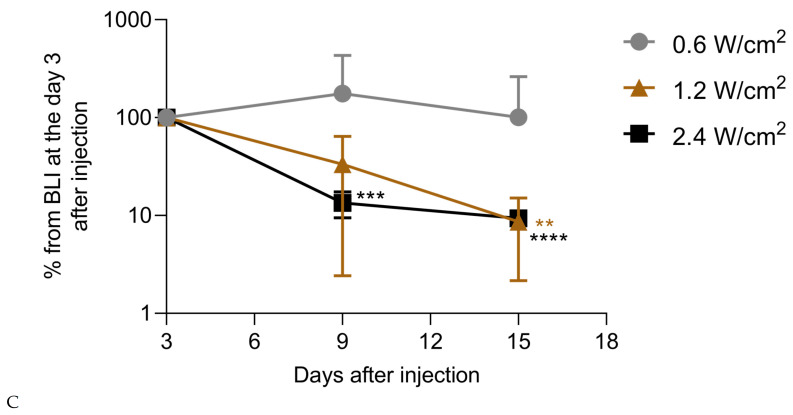

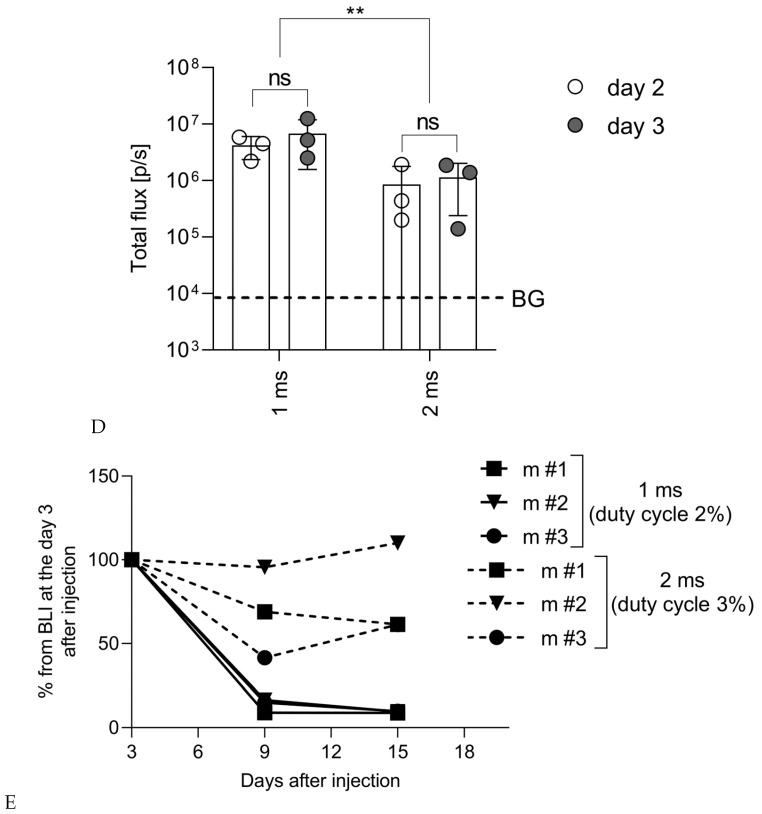
Expression of the Luc reporter introduced by id injection of pVaxLuc followed by sonoporation (id/SP) and monitored by BLI as photon flux from the injection sites (Series SP; Table 2). (**A**) Total photon flux from the injected sites sonoporated with acoustic intensities from 0.6 to 2.4 W/cm^2^ does not change between days 2 and 3 post-treatment. (**B**) Sonoporation at an acoustic intensity of 2.4 W/cm^2^ provides the highest total photon flux early after the treatment (days 2 to 3 pooled). (**C**) Changes of total photon flux from the injection sites with time (% of flux at day 3). (**D**) Total photon flux from the injected sites sonoporated with acoustic intensities of 2.4 W/cm^2^ and a pulse duration of 1 ms (duty cycle of 2%) or a pulse duration of 2 ms (duty cycle of 3%) does not change between days 2 and 3 post-treatment. (**E**) Change in total photon flux from the injection sites in individual mice (n = 3) sonoporated with a duty cycle of 2% and 3% (% of flux) at day 3. All data are presented as the mean bioluminescence level within each group ± SD. * *p* < 0.05, ** *p* < 0.01, *** *p* < 0.001, and **** *p* < 0.0001, ns—not significant (Kruskal–Wallis and Mann–Whitney tests and two-way ANOVA with Tukey correction for panel C). The background level of bioluminescence (BG) (dashed line) was set as the average signal from the area of the body equal to the ROI of naïve BALB/c mice (n = 3) injected with D-luciferin + SD.

Total photon flux (total flux) did not change from day 2 to day 3 (no significant change in the total flux was registered for the regime with acoustic intensity of either 0.6, 1.2, or 2.4 W/cm^2^; all *p* > 0.1) (Figure 2A), allowing us to pull flux data from these two days. Further comparison revealed that the early (days 2 to 3) signal from the injection sites sonicated with an acoustic intensity of 2.4 W/cm^2^ was significantly higher than that observed after sonication with lower acoustic intensities (Figure 2B). The follow-up of Luc reporter expression demonstrated a steady loss of signal in the group sonicated with an acoustic intensity of 2.4 W/cm^2^. By day 9, the loss was significantly higher than the loss of the signal in the groups sonicated with an acoustic intensity of 0.6 and 1.2 W/cm^2^ (Figure 2C), which could be explained by the immune-mediated elimination of Luc-expressing cells [40]. With this, the best results from the point of view of DNA immunization were achieved by id/SP with an acoustic intensity of 2.4 W/cm^2^.

Next, we assessed the effect of the value of duty cycle, testing the acoustic oscillation sequences of 1 ms (duty cycle 2%) and 2 ms (duty cycle 3%) at the optimal acoustic intensity of 2.4 W/cm^2^. Other parameters remained unchanged. In both regimens, total photon flux did not change from day 2 to day 3 (Figure 2D). On days 2–3, the pulled signal from the injection sites sonicated with a pulse with a duty cycle of 2% was significantly higher than that observed after sonication with a duty cycle of 3% (Figure 2D). After day 3, mice sonoporated with a duty cycle of 2% demonstrated a steady loss of bioluminescent signal from the id/SP sites compared to mice sonoporated with a duty cycle of 3% (Figure 2E).

For id-injected mice, gradual loss could be interpreted as a surrogate marker of the development of an anti-Luc immune response, exterminating expressing cells. This and the fact that reporter expression after 1 ms pulses (duty cycle 2%) was initially higher than after 2 ms pulses (duty cycle 3%) pointed to the advantage of the 1 ms SP regimen.

### 3.3. Optimization of Forced DNA Delivery by Electroporation: Efficient Delivery Is Determined by the Configuration of the Driving Pulse

Our next step was to compare the efficiency of id/SP delivery to that facilitated by the delivery “gold standard”, electroporation (id/EP). For this, we had to determine optimal EP conditions for our set of EP equipment, namely, the CUY21EDITII device combined with a multi-needle electrode. BALB/c mice were id-injected with pVaxLuc in an isotonic phosphate buffer (163 mmol/L) (as group ID.5; Table 1), and injection sites were subjected to EP using varying regimens (Series EP-1; Table 3).

**Figure 3:**
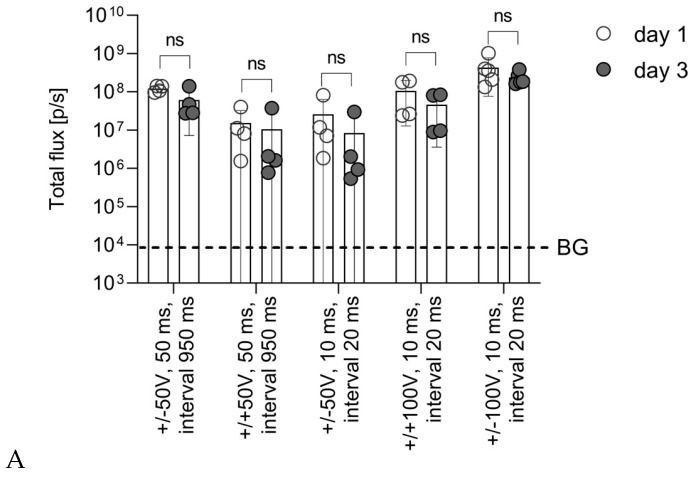

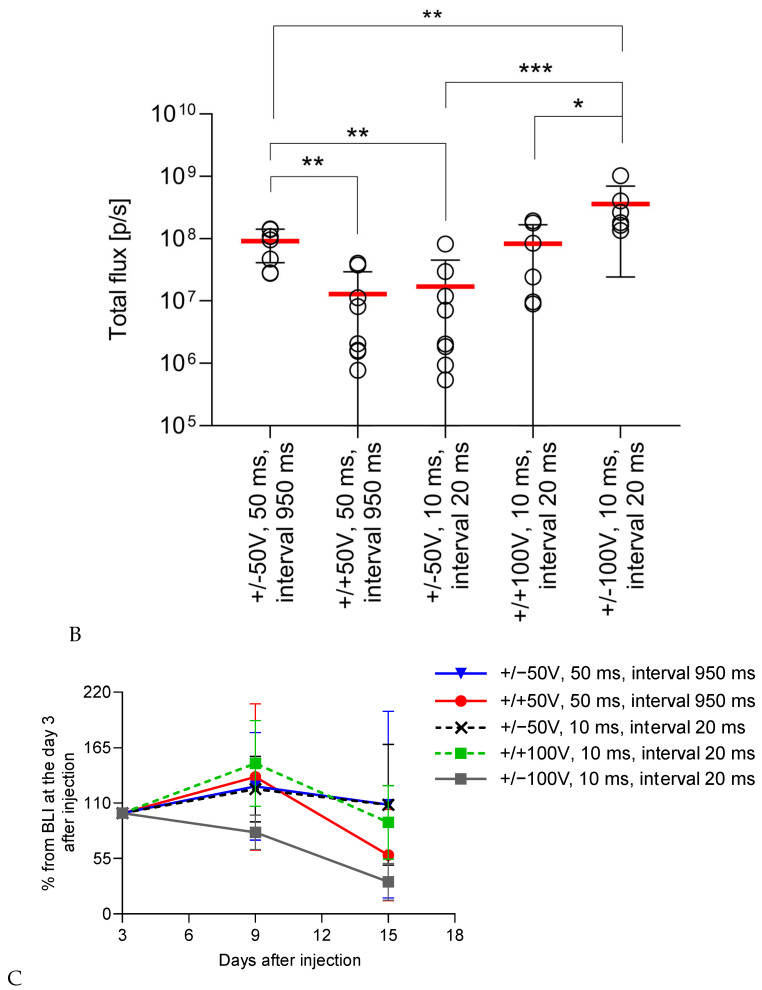
Expression of the Luc reporter after id injection of pVaxLuc in an isotonic phosphate buffer (163 mmol/L) (as group ID.5; Table 1) with subsequent electroporation (id/EP) and monitored by BLI as photon flux from the injection sites (Series EP-1 and EP-2; Table 3). (**A**) Stable levels of Luc reporter expression during the first three days after EP were demonstrated for all EP regimens (Series EP-1; Table 3). (**B**) Comparison of EP regimens in groups EP-1.1 to EP-1.5 by the levels of total photon flux secured on days 1 and 3 post-injection (pooled data from days 1 and 3 (**C**) and by the decrease in the levels of total photon flux from the injection sites as a % of the levels demonstrated on day 3. All data are presented as the mean bioluminescence level within each group ± SD. * *p* < 0.05, ** *p* < 0.01, and *** *p* < 0.001, ns—not significant (Kruskal–Wallis and Mann–Whitney tests). The background level of bioluminescence (BG) (dashed line) was set as the average signal from the area of the body equal to the ROI of naïve BALB/c mice (n = 3) injected with D-luciferin + SD.

Photon flux from the injection sites was monitored on day 1 and thereafter on days 3, 9, and 15 (as in the SP series). For all groups, total photon flux measured on day 1 and on day 3 did not differ (all *p* > 0.1) (Figure 3A). Data from these two days was pooled, and levels of reporter expression were compared. Comparison revealed that a regimen consisting of bipolar 50V pulses of 50 ms duration delivered with 950 ms intervals was superior to other 50V regimens, including those with high-frequency pulses (duration of 10 ms, interval of 20 ms) (Figure 3B). However, this regimen caused local burns and inflammation in the inter-electrode space, similar to the ones observed earlier in response to high long electric field pulsing conditions [54].

A way to increase efficacy, bypassing long pulses with long intervals, was to increase pulse intensity. Indeed, an increase in the voltage of the high-frequency pulses resulted in an increase in photon flux/reporter expression, specifically in the case of the bipolar pulses (Figure 3B). With this, the optimal EP protocol for the CUY21EDITII device equipped with a multi-needle electrode consisted of the high-frequency bipolar pulses of 100 V for 10 ms with an interval of 20 ms (Figure 3B).

As was mentioned above (Section 3.1), a gradual decrease in BLI after day 2 can serve as an indirect measure of the development of an anti-Luc immune response. Groups of mice demonstrated differences in the dynamics of bioluminescence from the third day after DNA delivery onwards (Figure 3C). A steady decrease was observed in mice receiving the bipolar pulses of 100 V for 10 ms with an interval of 20 ms (about 20% between days 3 and 9, and about 70% between days 3 and 15; *p* < 0.05), while no significant decrease was observed in the other groups (Figure 3C). A decrease observed in mice receiving the unipolar pulses of 100 V for 10 ms with an interval of 20 ms or unipolar pulses of 50 V for 10 ms with an interval of 950 ms was not statistically significant (*p* > 0.1 for both regimens) (Figure 2C). Thus, the regimen with a high voltage (100 V) and high-frequency pulses was superior to other regimens, both in terms of the initial levels of reporter expression and in the loss of signal with time, reflecting the extermination of expressing cells from the sites of injection.

Thus, the best results for id/EP DNA delivery were achieved using the EP regimen with bipolar driving pulses of 100 V with a duration of 10 ms and an interval of 20 ms between pulses. The regimen provided the highest levels of photon flux, manifesting the highest levels of Luc reporter expression, and a fast loss of bioluminescent signal after day 3 indicated immune clearance of expressing cells.

### 3.4. Intradermal Injection Facilitated by Sonoporation Is the Least Efficient Method of DNA Delivery

**Figure 4:**
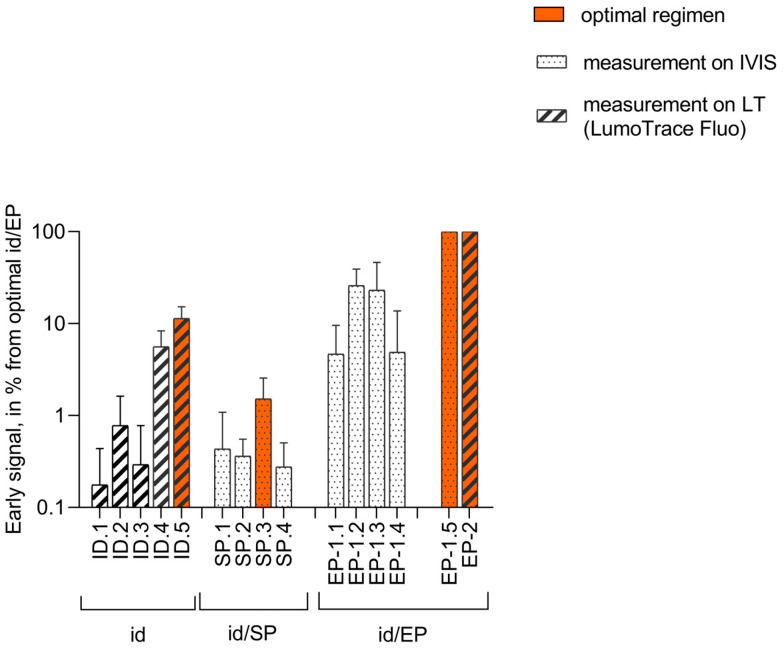
Comparison of DNA delivery by intradermal (id) injection alone, id injection forced by sonoporation (id/SP), or electroporation (id/EP) in the capacity to induce early expression of the Luc reporter. The graph compares deliveries by id (Series ID, groups ID.1 to ID.4 and ID.5 (optimal); Table 1) monitored by LumoTrace Fluo (LT), id/SP (Series SP, groups SP.1 to SP.4, SP.3 (optimal); Table 2) monitored by IVIS, and id/EP (Series EP, groups EP-1.1 to EP-1.4 and EP-1.5 (optimal); Table 3) monitored by IVIS. Bars representing the best DNA delivery protocol in ID, SP, and EP series are colored orange (ID.5, SP.3, and EP-1.5, respectively). Bars representing results assessed by IVIS are dotted, and those assessed by LT are striped. The results of the optimal DNA delivery by each of the methods are colored red. Total photon flux (IVIS) and total counts (LT) registered by the respective imager on day 2 after pVaxLuc delivery by optimal id/EP monitored were taken to be 100%. Percentages are calculated based on the mean total flux/total count values of all injection sites in the group at a given time point.

Next, we compared the levels of reporter expression after optimized DNA delivery facilitated by SP to those provided by EP to see if SP could provide matching results. Photon flux after id injection alone or id forced by SP (id/SP) was presented as a ratio to that of id/EP in the series monitored using one and the same device. Maximum photon flux was taken to be 100% after optimal id/EP delivery and was monitored with IVIS (EP-1) or LumoTrace Fluo (EP-2). Early (day 2–3) signals in the optimal ID delivery without EP and in the optimal id/SP were presented as a % of id/EP (SP.4 as a % of EP-1.5 for IVIS, and ID.4 as a % of EP-2 for LumoTrace Fluo). The efficiency of DNA delivery by id/SP was extremely low compared to that facilitated by EP (same plasmid dose) and after id alone in both the best and the second best regimens, although the latter comparison was confounded by id injections using a two-times higher dose of DNA. This indicated that early after the treatment (before the first manifestations of the adaptive immune response to Luc reporter), SP as a treatment had no positive effect on Luc reporter expression, which was comparable to the effect of EP (Figure 4).

We have also compared the degree of the loss of reporter signal starting from day 5, interpreting the loss as a surrogate marker of the development of adaptive immune response against luciferase. Comparison of the best regimens of DNA delivery by id/SP (SP.3, Figure 2E) and by id/EP (EP-1.5, Figure 3C) demonstrated that only the latter provided a steady decrease in the signal throughout the experiment. In the id/SP group, the signal was overall low (Figure 4) and also showed no significant change with time (Figure 2C,D), indicating that in the capacity to induce an immune response against luciferase, id/SP was inferior to the other two methods.

### 3.5. Comparison of DNA Delivery by Id Injection Without and with Electroporation in the Capacity to Induce an Immune Response in Mice

In the experiments described above, we have interpreted the loss of Luc reporter expression as a surrogate marker for the induction of an immune response against the luciferase. To give experimental support to this statement, we performed DNA immunization of mice with an immunogen capable of inducing a strong antibody response, namely, HIV-1 reverse transcriptase [55]. We have shown earlier that the consensus inactivated reverse transcriptase of HIV-1 clade A FSU_A strain (RT_A) is efficiently expressed from a synthetic expression optimized gene [41], but its immunogenicity as a DNA vaccine was not characterized. DNA immunization with enzymatically inactive RT_A (RT_Ain) was performed by id injection alone and id reinforced by EP. DNA immunization by id reinforced by SP was omitted from the immunogenicity tests due to the low efficacy of DNA delivery.

The best conditions chosen for delivery were reproduced and tested for immunogenicity (Series IR; Table 4). These conditions included id forced by EP (group EP-1.5; Table 3), two id delivery modes: the best one and another one found also effective (DNA in phosphate buffers with an ionic strength of 163 and 81 mmol/L; groups ID-1.4 and ID-1.5 respectively; Table 1). Additionally, for monitoring purposes, the poorest mode for the DNA delivery by id alone, DNA in the water (group ID-1.1; Table 1) was tested for immunogenicity. With this, we aimed to see how the difference in the delivery/expression would impact the magnitude of the immune response. A group of naive mice was used as a negative control (Series IR.5; Table 4). Mice were immunized with a prime on day 1, followed by a boost on day 21, as recommended [56]. On day 14 after the boost, mice were sacrificed, and total blood was collected and assessed for the levels of antibodies against the consensus RT of HIV-1 clade A strains FSU_A (anti-RT).

**Figure 5:**
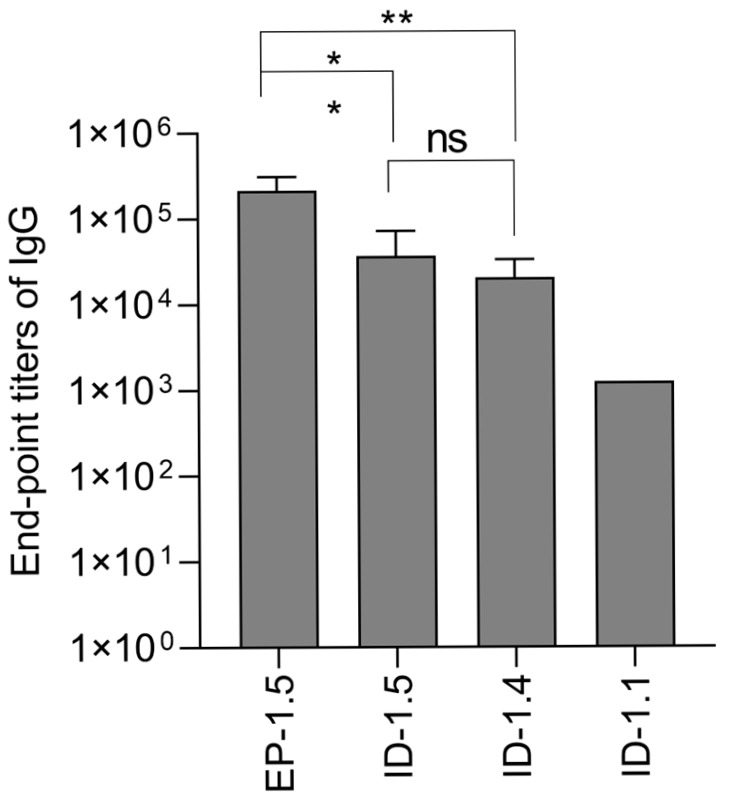
Average endpoint titers of IgG antibodies against consensus reverse transcriptase HIV-1 clade A FSU_A strain after prime–boost immunization of mice with plasmid pVaxRT_Ain delivered by an isotonic phosphate buffer (163 mmol/L) (ID-1.5; Table 1), a phosphate buffer with an ionic strength of 81 mmol/L (ID-1.4; Table 1), or deionized H_2_O (ID-1.1; Table 1), or by id injection in an isotonic phosphate buffer (163 mmol/L) followed by EP with bipolar 100V 10 ms pulses delivered with 20 ms intervals (EP-1.5) (Series IR; Table 4). * *p* < 0.05, ** *p* < 0.01 (Kruskal–Wallis, Mann–Whitney tests).

DNA immunization with pVaxRT_Ain in an isotonic phosphate buffer (163 mmol/L) followed by EP induced the average anti-RT antibody titers at a level of 2.1 × 10^5^ ± 9.6 × 10^4^) (Figure 5). DNA immunization with pVaxRT_Ain in a phosphate buffer with an ionic strength of 81 mmol/L and 163 mmol/L generated anti-RT IgG in the titer of up to 2 × 10^4^ ± 1.3 × 10^4^ and 4 × 10^4^ ± 3.6 × 10^4^, respectively. The latter was two times higher, but the difference did not reach the level of significance (Figure 5). In the group receiving pVaxRT_Ain in deionized H_2_O, anti-RT antibodies were only produced in one animal; their titer was 200 times lower than in mice DNA-immunized with RT_Ain by id/EP (1.2 × 10^3^ compared to 2.1 × 10^5^).

Our results show that the antibody response can be induced by id injection of plasmid DNA not only in an isotonic phosphate buffer (163 mmol/L) but also in a phosphate buffer with two times lower ionic strength. The total IgG titers in these groups did not differ. At the same time, the level of anti-RT antibodies induced by these regimens was 10-fold lower than the level of antibodies induced by id injection forced by electroporation (Figure 5). A tenfold difference in the magnitude of antibody levels (id/EP compared to id in an isotonic phosphate buffer in titers) reproduced the difference in the level of early expression of the Luc reporter by optimal id/EP compared to id alone (Figure 4). Strong anti-RT antibody response observed in mice DNA-immunized by id/EP corroborated the concept that the loss of protein (Luc reporter) expression after day 5 can indeed reflect the development of a specific immune response.

## 4. Discussion

Today, nearly 40 years after the discovery of DNA vaccines, their delivery into the cells is still a factor limiting their broad use. In vivo delivery of exogenous DNA is a non-trivial task. Plasmid DNA has to enter the cell, but as a hydrophilic, polar, and negatively charged molecule, it cannot freely cross the cellular membrane. DNA must additionally cross the nuclear envelope to the nucleus to be transcribed to mRNA to be translated into the immunogenic protein [35]. All DNA delivery methods include a chemical delivery step, i.e., the introduction of DNA solution by a needle, microneedle, sponge, liquid, or gas flow [15]. For this, DNA vaccines are dissolved in a buffer suitable for in vivo use, i.e., non-toxic and physiocompatible. The most common is phosphate-buffered saline (PBS) or saline, both providing an isotonic environment [15]. The use of PBS is preferable, as it stabilizes pH/prevents changes in the acidity, thus supporting normal operation of the cell. While PBS is isotonic, a solution of DNA in PBS cannot be considered truly isotonic. The components of PBS, NaCl, and KCl are strong electrolytes that completely dissociate into ions. Na^+^ and K^+^ are attracted to negatively charged phosphate groups and electronegative sites on DNA bases [57,58]. Binding neutralizes the repulsive forces between DNA strands, stabilizes the DNA duplex, and increases the efficiency of its passage through the cellular membrane [57,59]. Na^+^ ions can accumulate locally between DNA molecules, facilitating the condensation of DNA molecules [60]. Condensed DNA is less susceptible to enzymatic degradation and interacts better with cell membranes, allowing it to penetrate cells more effectively through passive transport [61]. At the same time, binding to DNA reduces the effective ionic strength in the solution; the actual ionic strength is determined by the remaining “free” ions. How this affects DNA delivery is not precisely known. Altogether, this makes the ionic strength of the DNA delivery buffer an important basic parameter to optimize in both forced and non-forced delivery of plasmid DNA.

DNA penetration into the cell was claimed to be improved by increasing the ionic strength of the solution [34]. However, high concentrations of positively charged ions change native DNA conformation, causing twisting [62]. Furthermore, an excessive absorption of monovalent ions inverts the effective DNA charge from negative to positive (overcharging), resulting in DNA destabilization [63]. Additionally, injection of DNA in the hypertonic solution can cause cell damage and inflammation [34,36]. With this, we only assessed the effects of the following lowering of ionic strength.

A hypotonic shock alone can cause temporary changes in the shape of the cell membrane, lasting up to five minutes [37]. During this time, particles up to 500 nm in size can be absorbed. When comparing the uptake of 100 and 500 nm nanoparticles by cultured cells under hypotonic and hypertonic conditions, it was shown that while a hypertonic environment did not significantly affect the efficiency of intracellular delivery, exposure of cells to hypotonic shock significantly increased the internalization efficiency of particles of any size [37]. This effect can be valuable in in vivo applications, but it needs to be used with caution, as excessive lowering of ionic strength will increase the repulsion between the phosphate groups of DNA, destabilize DNA complexes, lower the efficiency of delivery, and ultimately cause cell swelling and bursting (cell death).

In the first step of this study, we sought to determine the effect of lowering the ionic strength on the efficacy of delivery and a threshold of the ionic strength of the buffer at which DNA can still be effectively delivered by needle injection. To reveal the effect, we used high doses of DNA, 50 to 100 mcg per injection (2.5 to 5 mg/mL), to maximize the DNA-modulated decrease in the ionic strength of a delivery buffer in a non-facilitated DNA delivery (by id injection alone; Series ID; Table 1). We reasoned that lesser DNA amounts would produce a lesser effect, not visually translating into the efficacy of delivery and further into immunogenicity. Antigen expression and the level of immune response in DNA immunization were shown earlier to be dependent on DNA dose, in both experimental animals [64,65] and human volunteers [66,67], without signs of dose-dependent toxicity [67]. With this, we expected high DNA doses to result in high expression of the encoded protein and a strong immune response.

Reporter DNA delivered both in an isotonic phosphate buffer (163 mmol/L) and a phosphate buffer with an ionic strength of 81 mmol/L was well expressed, while deliveries at lower ionic strengths were equally ineffective. Furthermore, a significant loss of bioluminescent signal over time was observed after DNA injections in isotonic phosphate buffers and phosphate buffers with ionic strengths of 81 mmol/L, indicating the development of an immune response against Luc, while no decrease was observed after DNA delivery in buffers of lower ionic strengths. With this, a threshold of the ionic strength of the phosphate buffer at which DNA can still be effectively delivered by id injection was estimated to be 81 mmol/L. The absence of differences between a 41 mmol/L phosphate buffer and deionized water indicates that even in a diluted phosphate buffer with an ionic strength of 41 mmol/L all free Na^+^ and K^+^ ions were bound by DNA, as the delivery in this solution did not differ from the one in the water. This allows us to extrapolate that in the isotonic phosphate buffer containing 5 mg/mL of DNA, 25% of Na^+^ and K^+^ ions are bound by DNA, reducing the actual ionic strength to approx. 120 mmol/L, i.e., all deliveries in this study were actually performed in hypotonic solutions. These results are consistent with the previous studies demonstrating the positive effect of (mildly) hypotonic solutions on the delivery of DNA into cells both in vitro and in vivo in the context of nanoparticles [68,69].

DNA delivery by physical methods is much more effective than chemical delivery, with electroporation (EP) being a “golden standard” [70]. The parameters of EP depend on the type of laboratory animal, immunization site(s)/tissues, the antigen and vaccine composition, and also, importantly, the EP equipment, not allowing a universal EP protocol [20]. This drives continued optimization of the EP protocols, specifically, the parameters of electrical pulses and their shape, intensity, duration, frequency, and polarity [71,72,73,74,75]. These parameters jointly configure the changes caused by the pulse in the lipid membranes of cells and have to be optimized in combination to ensure efficient DNA delivery. In this study, we optimized EP parameters to determine an optimum and further used them as a reference for other methods of DNA delivery. We performed EP as a two-stage process involving one high-voltage poration pulse and eight low-voltage square-shaped pulses, which were repeatedly shown to be highly effective [76,77,78]. Based on the experiments determining the efficacy of DNA delivery by id injection alone, showing an ionic strength of ≥82 mmol/L as the best, and considering the dependence of EP on buffer conductivity, we delivered Luc reporter DNA in an isotonic phosphate buffer. Parameters that varied were pulse duration, interval between the pulses, pulse intensity, and polarity.

The duration of the electrical pulse and repetition rate (interval between the pulses) determine the formation of pores and, through this, penetration of DNA into the cell. Earlier, in in vitro cell transfection, it was shown that 100 microsecond pulses created only small, quickly closing pores, which allowed entry into the cell of the molecules of the size of sucrose (molecular mass of 342.3 Da, radius of 0.44 to 0.52 nm) [79]. Pulses >2 ms forming pores surviving for 15–20 min were recommended [79]. Another important parameter is the interval between the pulses. Both short (<5 ms) and long lags (>100 s) decrease the efficacy of EP [80]. Based on these findings, we have chosen the millisecond pulses and tried two regimens, a “long” one with 50 ms pulses with a 950 ms (16 s) interval and a “short” one with 10 ms pulses with a 20 ms interval (both at 50 V). As could be expected, longer pulses with lower repetition frequencies led to more efficient electroporation, resulting in higher levels of Luc expression. However, the “long” regimen was traumatic, causing local burns.

To increase EP efficiency, we increased pulse intensity, or electric field strength (V/m). The length was pre-determined by the distance between the electrodes in the multi-needle DERMAVAX electrode; the parameter to change was the voltage. For a non-traumatic “short” regimen, an increase in pulse voltage from 50 to 100 V led to a tenfold increase in the photon flux, indicating orders of magnitude higher levels of Luc expression. Importantly, the “short” regimen consisting of 100V pulses was more efficient than the “long” regimen consisting of 50 V pulses.

Another parameter that varied was pulse polarity. The latter modulates the restructuring of lipid membranes of the cells and shapes the area (zone) of DNA–cell membrane interaction [81]. There are also other factors contributing to DNA delivery and reporter expression. In electric cell fusion experiments, bipolar pulses were reported to cause less cell death [82,83]. For single cells, over ten times more bipolar pulses were required to induce cell death compared to monopolar pulses of similar magnitude and duration [83]. The effect was attributed to bipolar cancellation, a phenomenon in which the application of the second electric pulse reduces (“cancels”) cell membrane damage by a preceding electric pulse of the opposite polarity [84]. Additionally, the concentration of metal ions released from the electrodes by bipolar pulses is more than an order of magnitude lower than that released by unipolar pulses of the same amplitude and duration [74,85]. Due to this, bipolar pulses cause less electrolytic contamination of the cells. In our study as well, both “short” and “long” regimens of bipolar configuration resulted in higher photon flux indicating higher levels of Luc expression compared to the monopolar pulses of the same configuration. Altogether, these allowed us to select a “short” regimen with 100 V bipolar pulses as the optimal and use it as a reference in evaluation of the efficacy of other methods of DNA delivery.

An attractive method of physical delivery is sonoporation (SP). Compared to electroporation, this method is more accessible and less traumatic. The effectiveness of SP has not been directly compared to EP. Here, we sought to optimize SP and further compare its performance to that of optimized EP. The dynamic characteristics of the membrane pores formed by SP depend on the level of acoustic pressure or the intensity of acoustic vibrations [86,87], the duration of sonication [30], and the percentage of the active time of a periodic signal relative to its period (the duty cycle) [28]. A smaller value of the duty cycle preserves cell viability, but it can reduce the transfection efficiency [28]. In addition to the cavitation, SP can induce local heating. High-intensity ultrasound can cause an increase in the tissue temperature and enhance the permeability of the cell membrane [88]. However, it can also cause cell destruction, motivating minimization of thermal effects [89]. With this, the parameters we varied to ensure effective intracellular delivery by SP included the acoustic pressure (ultrasound intensity) and insonification duration/duty cycle.

Cavitation, which occurs in the liquid medium of the intercellular space under the influence of ultrasound, is the primary mechanism that facilitates the formation of pores in the plasma membrane. Studies that tested SP as a method for DNA delivery into cells relied on enhancing cavitation by introducing the “exogenous” microbubbles instead of increasing the number of natural ones by increasing the intensity of acoustic waves. With the use of “exogenous” microbubbles, the process would rely only on the capacity of sonic waves to burst these added bubbles and make holes in cell membranes. An increased impact on the cell membrane (pore formation) can, thus, be reached by increasing the number of added microbubbles (without the necessity to increase the intensity of acoustic waves). However, a balance must be maintained, as exceeding the threshold level of microjet exposure from the additional “exogenous” microbubbles will cause irreversible damage to cell membranes and cell death by lysis. With this, the use of “exogenous“ microbubbles introduces an additional parameter to optimize, namely, the optimal microbubble composition, size, and dose, a combination of parameters specific for the tissues to be targeted by sonication (see, for example, [90,91]). This, together with the dependence of the method on the provision of microbubbles and related extra costs, adds limitations to the use of “exogenous” microbubble-based SP. There are also issues of histocompatibility and toxicity of the “exogenous” microbubbles, as well as the potential harm caused by the long-term accumulation of the breakdown products of “exogenous” microbubbles in the body [25]. Considering these aspects, in the present study, we deliberately withheld from enhancing cavitation with “exogenous” microbubbles. In direct comparison of the effectiveness of id, id/SP, and id/EP delivery, DNA was delivered in an isotonic phosphate buffer.

Based on the high level of bioluminescent signal from the plasmid DNA injection sites, we have shown that increasing the acoustic intensity, or acoustic pressure, to 2.4 W/cm^2^ increased the efficiency of tissue DNA delivery, with a 2% duty cycle being optimal. However, even when optimized, this method was much less effective than EP. Moreover, if not used with microbubbles, the method was less effective than id DNA injection alone. Low efficiency could be listed in part, attributed to an increased cell death due to thermal effects (local heating) [88] and destruction and/or of the cellular cytoskeleton [89]. Due to the extremely low efficiency of DNA delivery using SP, even after optimization, this method was not evaluated for its capacity to induce an immune response.

In the final stage, we compared DNA delivery by id injection with and without electroporation for the capacity to induce an immune response in mice and also checked the effect on immunogenicity of the reduction in the ionic strength of the DNA delivery buffer. As a DNA immunogen, we have chosen HIV-1 reverse transcriptase (RT) due to its ability to induce a strong antibody response in mice [92]. We found that RT DNA delivered in phosphate buffer with an ionic strength of 81 mmol/L induced an antibody response of the same magnitude as DNA delivered in an isotonic phosphate buffer with an ionic strength of 163 mmol/L, whereas no antibody response was induced if RT DNA was delivered in deionized water. Notably, antibody titers induced after id/EP differed from antibody titers induced by id injection of DNA by ten times, as did the levels of expression of the reporter protein delivered by id and by id/EP.

Taken together, our data indicate that (i) the ionic strength of the phosphate buffer of 81 mmol/L is a threshold beyond which the expression of id-injected DNA was still supported; (ii) EP facilitate DNA delivery/expression of delivered DNA at least 10-fold compared to id injection alone; (iii) SP not assisted by microbubbles does not facilitate DNA delivery; furthermore, it reduces expression of genes delivered by id injection; and (iv) both methods of DNA delivery, id and id/EP, induce an antibody response, with differences in the antibody levels reached by each of the methods proportional to the differences in the levels of protein expression.

## 5. Conclusions

The efficacy of id DNA delivery strongly depends on the ionic strength of the buffer used to dissolve DNA. The ionic strength of 81 mmol/L of phosphate buffer is a threshold at which gene expression after id DNA injection is still supported. Variations in the ionic strength of the delivery buffer within the interval of 81 to 164 mmol/L do not affect the magnitude of the humoral response. Id injection reinforced by sonoporation (id/SP), without the use of microbubbles, is unable to promote effective DNA delivery. Both the levels of antigen expression provided by id injection reinforced by electroporation (id/EP) injection and the levels of antibodies are orders of magnitude higher than the levels provided by id injection alone. Thus, after optimization of three delivery methods, id, id/SP (without microbubbles), and id/EP, the latter generates the best results with respect to the level of exogenous/reporter protein expression and the consecutive immune (antibody) response to the foreign antigen.

## 6. Limitations

This study used high doses of DNA (50 to 100 µg per injection site) to maximize the DNA-modulated decrease in the ionic strength of the delivery buffer [93]. A drawback of high DNA dose is the high viscosity of the solution interfering with the injection, with a potential risk of shearing of DNA while passing through the needle. Indeed, DNA can be sheared while passing through the small orifice of the hypodermic needle [94]. The process of hydrodynamic shearing employs Hamilton syringes with an inner diameter of 1.016 mm (0.04″). In the shearing process, DNA is driven through the needle by a pump at a relatively high flow rate (0.5 mL/min; [95]). In this study, injections of DNA were performed using insulin needles with a considerably larger inner diameter (29 G; 0.184 mm). Also, the flow rate during the manual injection was 10 times lower (injection of 20 mcl DNA solution took at least 30 s). With larger needles and a much slower passage rate, the risks of DNA shearing during the process of DNA immunization were perceived as low.

One of the limitations of the study was the use of varying DNA doses (50 and 100 mcg). In the first ID series, each mouse received two injections with a DNA dose of 100 mcg per injection. In this series, we acted on the assumption of low expression levels of Luc after unfacilitated injection and aimed to increase the chances of signal detection by increasing the DNA dose to the maximum that could still be delivered intradermally (volume limitation). As mentioned above, we faced difficulties in delivering this high dose in a comparatively low volume due to the high viscosity of the solution, and there was a risk of DNA fragmentation. In view of this, in all subsequent experiments with facilitated DNA delivery, where we expected an increase in the levels of Luc gene expression after sonoporation or electroporation, the dose of DNA was decreased from 100 to 50 mcg. In the case of EP, this approach worked well, as the bioluminescence signal generated after delivery of 50 mcg of Luc DNA was strong and could be reliably detected. In the case of SP, the result was not as good as we expected; SP did not lead to an improvement in Luc gene delivery and a subsequent increase in Luc expression. BLI signals were low. Since SP was not shown to improve Luc expression after ID delivery, a decision was made not to repeat the experiment with higher DNA doses.

Another limitation was the use of varying numbers of DNA injections per animal (one or two). In case of non-facilitated delivery of the Luc gene, an increasing number of injection sites allowed for an increase in the number of observations without increasing the number of animals, which answered the general 3R requirements for animal research. We exploited this option in the delivery of the Luc gene in the ID series (five mice per group, generating 10 observations). All other experiments included subsequent physical treatment of the animal, by sonoporation or electroporation. Both treatments could affect the animal, causing local inflammation, such as swelling for SP and local burns for EP, which may affect tissues neighboring the injection site, including the site of the second injection (in a mouse body, two injection sites are separated by max 2–2.5 cm). Local inflammation would affect the results of experiments assessing the levels of gene expression, which grossly rely on the minimal interference of the innate immune response. Based on these considerations, all experiments with facilitated ID injections were performed at one site per mouse. The only exclusion was the experiment EP-2 in the EP series, where DNA was injected into two sites with subsequent electroporation. This exclusion was made to be able to compare the results of ID delivery alone (performed at two sites) with ID delivery facilitated by electroporation.

Importantly, despite variations in the plasmid DNA dose and in the number of injections per animal, we were able to reliably show that id/EP is the most efficient method for both the delivery of DNA/foreign protein expression and for the induction of the immune response against the encoded DNA immunogen, although differences in the dose of injected DNA complicated the throughout comparison of the study results.

Some experiments, such as delivery by id/SP, were performed in a pilot series of three mice and were not repeated on the larger groups of animals. In deliveries with questionable efficacy, we kept the number of animals in the experimental groups as low as possible to follow 3R principles (reduce, refine, replace). Larger groups could have produced the same results but with higher statistical significance.

For technical reasons, due to the performance of the experiments in two separate facilities, without the possibility of transferring the animals, monitoring the expression of luciferase by bioluminescent imaging was performed on two different imagers: IVIS (Perkin Elmer) and LucoTrace Fluo (Abisense). Differences in the acquisition and quantification of the photon emission were mitigated by the performance of one and the same experiment (id/EP delivery) in two repeats, with assessment of the results by IVIS (EP-1.5) and by LumoTrace Fluo (EP-2), and further normalization of the results of other experiments to the “golden standard” assessed by the respective assessment technique.

Delivery by id/SP of DNA immunogen encoding HIV-1 RT was omitted from the DNA immunization series due to the inefficacy of this delivery method. It would have been good to perform this experiment to see how weak the immune response would be in comparison to that in id alone and id with EP.

Overall, the limitations were technical and had no major effects on the main results and conclusions of this study.

## Figures and Tables

**Table 1:** A series of experiments in BALB/c mice performed to determine the threshold ionic strength of a phosphate buffer supporting the delivery of plasmid DNA encoding luciferase (pVaxLuc) by id injection and assessed by in vivo expression of the Luc reporter using in vivo bioluminescent imaging (BLI) on LumoTrace Fluo (Abisense, Moscow, Russia) (see Section 2.8 for details).

Group * (nn Mice Per Group)	Number of Injection Sites	Dose of DNA Per Injection, μg	Ionic Strength of Phosphate Buffer Used to Dissolve DNA, mmol/L **
ID.1 (n = 5)	2	100	0 (deionized water H_2_O)
ID.2 (n = 5)	2	100	20
ID.3 (n = 5)	2	100	41
ID.4 (n = 5)	2	100	81
ID.5 (n = 5)	2	100	163

*—the group code includes the name of the experimental series and the group number, separated by a period. **—not counting the effect on the ionic strength of DNA molecules.

**Table 2:** A series of experiments in BALB/c mice performed to optimize the reinforcement of DNA delivery by sonoporation after initial id injection of plasmid DNA encoding luciferase (pVaxLuc) in a phosphate buffer with an ionic strength of 163 mmol/L, with efficacy assessed by in vivo expression of the Luc reporter using in vivo BLI on IVIS (Perkin Elmer, Waltham, MA, USA) (see Section 2.8 for details).

Group * (nn Mice Per Group)	Number of Injection Sites	Dose of DNA Per Injection, µg	Variable Pulse Parameters of Ultrasound	
			Pulse Duration, ms	Acoustic Intensity, W/cm^2^
SP.1 (n = 3)	1	50	1	0.6
SP.2 (n = 3)	1	50	1	1.2
SP.3 (n = 3)	1	50	1	2.4
SP.4 (n = 3)	1	50	2	2.4

*—the group code includes the name of the experimental series and the group number, separated by a period.

**Table 3:** A series of experiments in BALB/c mice performed to optimize the reinforcement of DNA delivery by electroporation after initial id injection of plasmid DNA encoding luciferase (pVaxLuc) in an isotonic phosphate buffer (163 mmol/L), with efficacy assessed by in vivo expression of the Luc reporter using in vivo BLI on IVIS (Perkin Elmer, Waltham, MA, USA) (Series EP-1) or LumoTrace Fluo (Abisense, Moscow, Russia) (Series EP-2) (see Section 2.8 for details).

Group * (nn Mice Per Group)	Number of Injection Sites	Dose of DNA Per Injection, µg	Parameters of the Driving Pulses			
			Voltage, V	Polarity, Constant (+/+)/ Variable (+/−)	Pulse Duration, ms	Interval Between Pulses, ms
EP-1.1 (n = 4)	1	50	50	+/+	50	950
EP-1.2 (n = 4)	1	50	50	+/−	50	950
EP-1.3 (n = 4)	1	50	50	+/−	10	20
EP-1.4 (n = 4)	1	50	100	+/+	10	20
EP-1.5 (n = 4)	1	50	100	+/−	10	20
EP-2 (n = 3)	2	100	100	+/−	10	20

*—the group code includes the name of the experimental series and the group number, separated by a period (for Series EP-1), or only the name of the experimental series (for Series EP-2).

**Table 4:** A series of experiments in BALB/c mice performed to determine the efficacy of immune response to inactivated HIV-1 reverse transcriptase induced by the delivery of encoding plasmid pVaxRT_Ain by id injections in a buffer of varying ionic strength with or without electroporation.

Group * (nn Mice Per Group)	Number of Injection Sites	Dose of DNA Per Injection, µg	Ionic Strength of Phosphate Buffers Used to Dissolve DNA, mmol/L **
IR.1 (n = 5)	1	100	0 (deionized H_2_O)
IR.2 (n = 5)	1	100	81
IR.3 (n = 5)	1	100	163
IR.4 *** (n = 4)	1	100	163
IR.5 (n = 3)	0	0	naïve mice

*—the group code includes the name of the experimental series and the group number, separated by a period. **—not counting the effect on the ionic strength of DNA molecules. ***—the group was electroporated with the regimen found to be optimal in Series EP-1 (EP-1.5; Table 3).

## Data Availability

Data is contained within the article.

## References

[1] Khobragade A., Bhate S., Ramaiah V., Deshpande S., Giri K., Phophle H., Supe P., Godara I., Revanna R., Nagarkar R. (2022). Efficacy, Safety, and Immunogenicity of the DNA SARS-CoV-2 Vaccine (ZyCoV-D): The Interim Efficacy Results of a Phase 3, Randomised, Double-Blind, Placebo-Controlled Study in India. Lancet.

[2] Lopes A., Vandermeulen G., Préat V. (2019). Cancer DNA Vaccines: Current Preclinical and Clinical Developments and Future Perspectives. J. Exp. Clin. Cancer Res..

[3] Li L., Saade F., Petrovsky N. (2012). The Future of Human DNA Vaccines. J. Biotechnol..

[4] Kutzler M.A., Weiner D.B. (2008). DNA Vaccines: Ready for Prime Time?. Nat. Rev. Genet..

[5] Leitner W.W., Ying H., Restifo N.P. (1999). DNA and RNA-Based Vaccines: Principles, Progress and Prospects. Vaccine.

[6] Klinman D.M., Barnhart K.M., Conover J. (1999). CpG Motifs as Immune Adjuvants. Vaccine.

[7] Pushko P., Ishmukhametov A.A., Bredenbeek P.P., Lukashevich I.S. (2019). Experimental DNA-Launched Live-Attenuated Vaccines Against Yellow Fever. Epidemiol. Vaccinal Prev..

[8] Fanelli A., Mantegazza L., Hendrickx S., Capua I. (2022). Thermostable Vaccines in Veterinary Medicine: State of the Art and Opportunities to Be Seized. Vaccines.

[9] Kisakov D.N., Orlova L.A., Sharabrin S.V., Rudometov A.P., Borgoyakova M.B., Starostina E.V., Karpenko L.I., Ilyichev A.A. (2022). Delivery of a DNA Vaccine Encoding SARS-CoV-2 Receptor-Binding Domain (RBD) by Electroporation. Med. Acad. J..

[10] Du X., Wang J., Zhou Q., Zhang L., Wang S., Zhang Z., Yao C. (2018). Advanced Physical Techniques for Gene Delivery Based on Membrane Perforation. Drug Deliv..

[11] Lim M., Badruddoza A.Z.M., Firdous J., Azad M., Mannan A., Al-Hilal T.A., Cho C.-S., Islam M.A. (2020). Engineered Nanodelivery Systems to Improve DNA Vaccine Technologies. Pharmaceutics.

[12] Ledesma-Feliciano C., Chapman R., Hooper J.W., Elma K., Zehrung D., Brennan M.B., Spiegel E.K. (2023). Improved DNA Vaccine Delivery with Needle-Free Injection Systems. Vaccines.

[13] Li Z., Zhang L., Jiang K., Zhang Y., Liu Y., Hu G., Song J. (2022). Biosafety Assessment of Delivery Systems for Clinical Nucleic Acid Therapeutics. Biosaf. Health.

[14] Weniger B.G., Papania M.J. (2013). Alternative Vaccine Delivery Methods. Vaccines.

[15] Wang S., Lu S. (2013). DNA Immunization. Curr. Protoc. Microbiol..

[16] Peng S., Fan D., Tu H.-F., Cheng M., Arend R.C., Levinson K., Tao J., Roden R.B.S., Hung C.-F., Wu T.-C. (2024). Improved Efficacy of Therapeutic HPV DNA Vaccine Using Intramuscular Injection with Electroporation Compared to Conventional Needle and Needle-Free Jet Injector Methods. Cell Biosci..

[17] Puri N., Weyand E.H., Abdel-Rahman S.M., Sinko P.J. (2000). An Investigation of the Intradermal Route as an Effective Means of Immunization for Microparticulate Vaccine Delivery Systems. Vaccine.

[18] Ito K., Ito K., Shinohara N., Kato S. (2003). DNA Immunization via Intramuscular and Intradermal Routes Using a Gene Gun Provides Different Magnitudes and Durations on Immune Response. Mol. Immunol..

[19] Pirc E., Balosetti B., Miklavčič D., Reberšek M. (2020). Electronic Emulator of Biological Tissue as an Electrical Load during Electroporation. Appl. Sci..

[20] Kisakov D.N., Belyakov I.M., Kisakova L.A., Yakovlev V.A., Tigeeva E.V., Karpenko L.I. (2024). The Use of Electroporation to Deliver DNA-Based Vaccines. Expert Rev. Vaccines.

[21] Daftarian P., Chowdhury R., Ames P., Wei C., King A.D., de Rivero Vaccari J.P., Dillon L., Price J., Leung H., Ashlock B. (2011). In Vivo Electroporation and Non-Protein Based Screening Assays to Identify Antibodies against Native Protein Conformations. Hybridoma.

[22] Liu W.-W., Liu S.-W., Liou Y.-R., Wu Y.-H., Yang Y.-C., Wang C.-R.C., Li P.-C. (2016). Nanodroplet-Vaporization-Assisted Sonoporation for Highly Effective Delivery of Photothermal Treatment. Sci. Rep..

[23] Miller D.L., Pislaru S.V., Greenleaf J.E. (2002). Sonoporation: Mechanical DNA Delivery by Ultrasonic Cavitation. Somat. Cell Mol. Genet..

[24] Blatteau J.-E., Souraud J.-B., Gempp E., Boussuges A. (2006). Gas Nuclei, Their Origin, and Their Role in Bubble Formation. Aviat. Space Environ. Med..

[25] He J., Liu Z., Zhu X., Xia H., Gao H., Lu J. (2022). Ultrasonic Microbubble Cavitation Enhanced Tissue Permeability and Drug Diffusion in Solid Tumor Therapy. Pharmaceutics.

[26] Oberli M.A., Schoellhammer C.M., Langer R., Blankschtein D. (2014). Ultrasound-Enhanced Transdermal Delivery: Recent Advances and Future Challenges. Ther. Deliv..

[27] Shi Y., Weng W., Chen M., Huang H., Chen X., Peng Y., Hu Y. (2023). Improving DNA Vaccination Performance through a New Microbubble Design and an Optimized Sonoporation Protocol. Ultrason. Sonochem..

[28] Pan H., Zhou Y., Izadnegahdar O., Cui J., Deng C.X. (2005). Study of Sonoporation Dynamics Affected by Ultrasound Duty Cycle. Ultrasound Med. Biol..

[29] Du M., Li Y., Chen Z. (2022). Sonoporation-Mediated Gene Transfection: A Novel Direction for Cell Reprogramming In Vivo. Front. Bioeng. Biotechnol..

[30] Shapiro G., Wong A.W., Bez M., Yang F., Tam S., Even L., Sheyn D., Ben-David S., Tawackoli W., Pelled G. (2016). Multiparameter Evaluation of in Vivo Gene Delivery Using Ultrasound-Guided, Microbubble-Enhanced Sonoporation. J. Control. Release.

[31] Lyons B., Balkaran J.P.R., Dunn-Lawless D., Lucian V., Keller S.B., O’Reilly C.S., Hu L., Rubasingham J., Nair M., Carlisle R. (2023). Sonosensitive Cavitation Nuclei—A Customisable Platform Technology for Enhanced Therapeutic Delivery. Molecules.

[32] Fan Z., Kumon R.E., Deng C.X. (2014). Mechanisms of Microbubble-Facilitated Sonoporation for Drug and Gene Delivery. Ther. Deliv..

[33] Wang M., Zhang Y., Cai C., Tu J., Guo X., Zhang D. (2018). Sonoporation-Induced Cell Membrane Permeabilization and Cytoskeleton Disassembly at Varied Acoustic and Microbubble-Cell Parameters. Sci. Rep..

[34] Chesnoy S., Huang L. (2002). Enhanced Cutaneous Gene Delivery Following Intradermal Injection of Naked DNA in a High Ionic Strength Solution. Mol. Ther..

[35] Kozak M., Hu J. (2024). DNA Vaccines: Their Formulations, Engineering and Delivery. Vaccines.

[36] Ensign L.M., Hoen T.E., Maisel K., Cone R.A., Hanes J.S. (2013). Enhanced Vaginal Drug Delivery through the Use of Hypotonic Formulations That Induce Fluid Uptake. Biomaterials.

[37] Ruzzante B., Fruzzetti F., Cattaneo M., Lauria Pinter G., Marcuzzo S., Candiani G., Bono N. (2025). Harnessing Osmotic Shock for Enhanced Intracellular Delivery of (Nano)Cargos. Int. J. Pharm..

[38] Zheng S., Li Y., Shao Y., Li L., Song F. (2024). Osmotic Pressure and Its Biological Implications. Int. J. Mol. Sci..

[39] Starodubova E., Krotova O., Hallengärd D., Kuzmenko Y., Engström G., Legzdina D., Latyshev O., Eliseeva O., Karin Maltais A., Tunitskaya V. (2012). Cellular Immunogenicity of Novel Gene Immunogens in Mice Monitored by In Vivo Imaging. Mol. Imaging.

[40] Petkov S.P., Heuts F., Krotova O.A., Kilpelainen A., Engström G., Starodubova E.S., Isaguliants M.G. (2013). Evaluation of Immunogen Delivery by DNA Immunization Using Non-Invasive Bioluminescence Imaging. Hum. Vaccines Immunother..

[41] Bayurova E., Jansons J., Skrastina D., Smirnova O., Mezale D., Kostyusheva A., Kostyushev D., Petkov S., Podschwadt P., Valuev-Elliston V. (2019). HIV-1 Reverse Transcriptase Promotes Tumor Growth and Metastasis Formation via ROS-Dependent Upregulation of Twist. Oxid. Med. Cell. Longev..

[42] Charan J., Kantharia N.D. (2013). How to Calculate Sample Size in Animal Studies?. J. Pharmacol. Pharmacother..

[43] Humphrey V.F. (2007). Ultrasound and Matter—Physical Interactions. Prog. Biophys. Mol. Biol..

[44] Datta S., Coussios C.-C., Ammi A.Y., Mast T.D., de Courten-Myers G.M., Holland C.K. (2008). Ultrasound-Enhanced Thrombolysis Using Definity as a Cavitation Nucleation Agent. Ultrasound Med. Biol..

[45] Fan Z., Chen D., Deng C.X. (2013). Improving Ultrasound Gene Transfection Efficiency by Controlling Ultrasound Excitation of Microbubbles. J. Control. Release.

[46] Shin H.-C., Prager R., Gomersall H., Kingsbury N., Treece G., Gee A. (2010). Estimation of Average Speed of Sound Using Deconvolution of Medical Ultrasound Data. Ultrasound Med. Biol..

[47] Soorani M., Anjani Q.K., Larrañeta E., Donnelly R.F., Das D.B. (2024). Modelling Insertion Behaviour of PVP (Polyvinylpyrrolidone) and PVA (Polyvinyl Alcohol) Microneedles. Int. J. Pharm..

[48] Chen N., Garner A.L., Chen G., Jing Y., Deng Y., Swanson R.J., Kolb J.F., Beebe S.J., Joshi R.P., Schoenbach K.H. (2007). Nanosecond Electric Pulses Penetrate the Nucleus and Enhance Speckle Formation. Biochem. Biophys. Res. Commun..

[49] Osorio F.G., De La Rosa J., Freije J.M. (2013). Luminescence-Based in Vivo Monitoring of NF-κB Activity through a Gene Delivery Approach. Cell Commun. Signal.

[50] Yu W.H., Moore D., Debs R.J., Kashani-Sabet M., Liggitt D., Heath T.D. (1999). Topical Gene Delivery to Murine Skin. J. Investig. Dermatol..

[51] Podetz-Pedersen K.M., Vezys V., Somia N.V., Russell S.J., McIvor R.S. (2014). Cellular Immune Response against Firefly Luciferase after Sleeping Beauty-Mediated Gene Transfer In Vivo. Hum. Gene Ther..

[52] Kupper T.S., Fuhlbrigge R.C. (2004). Immune Surveillance in the Skin: Mechanisms and Clinical Consequences. Nat. Rev. Immunol..

[53] Troy T., Jekic-McMullen D., Sambucetti L., Rice B. (2004). Quantitative Comparison of the Sensitivity of Detection of Fluorescent and Bioluminescent Reporters in Animal Models. Mol. Imaging.

[54] Pedron-Mazoyer S., Plouët J., Hellaudais L., Teissie J., Golzio M. (2007). New Anti Angiogenesis Developments through Electro-Immunization: Optimization by in Vivo Optical Imaging of Intradermal Electrogenetransfer. Biochim. Biophys. Acta (BBA)-Gen. Subj..

[55] Latanova A., Petkov S., Kuzmenko Y., Kilpeläinen A., Ivanov A., Smirnova O., Krotova O., Korolev S., Hinkula J., Karpov V. (2017). Fusion to Flaviviral Leader Peptide Targets HIV-1 Reverse Transcriptase for Secretion and Reduces Its Enzymatic Activity and Ability to Induce Oxidative Stress but Has No Major Effects on Its Immunogenic Performance in DNA-Immunized Mice. J. Immunol. Res..

[56] Latanova A.A., Petkov S., Kilpelainen A., Jansons J., Latyshev O.E., Kuzmenko Y.V., Hinkula J., Abakumov M.A., Valuev-Elliston V.T., Gomelsky M. (2018). Codon Optimization and Improved Delivery/Immunization Regimen Enhance the Immune Response against Wild-Type and Drug-Resistant HIV-1 Reverse Transcriptase, Preserving Its Th2-Polarity. Sci. Rep..

[57] Singh A., Maity A., Singh N. (2022). Structure and Dynamics of dsDNA in Cell-like Environments. Entropy.

[58] Cheng Y., Korolev N., Nordenskiöld L. (2006). Similarities and Differences in Interaction of K^+^ and Na^+^ with Condensed Ordered DNA. A Molecular Dynamics Computer Simulation Study. Nucleic Acids Res..

[59] Lipfert J., Doniach S., Das R., Herschlag D. (2014). Understanding Nucleic Acid-Ion Interactions. Annu. Rev. Biochem..

[60] Yoo J., Aksimentiev A. (2016). The Structure and Intermolecular Forces of DNA Condensates. Nucleic Acids Res..

[61] Teif V.B., Bohinc K. (2011). Condensed DNA: Condensing the Concepts. Prog. Biophys. Mol. Biol..

[62] Cruz-León S., Vanderlinden W., Müller P., Forster T., Staudt G., Lin Y.-Y., Lipfert J., Schwierz N. (2022). Twisting DNA by Salt. Nucleic Acids Res..

[63] Zhang C., Tian F.-J., Zuo H.-W., Qiu Q.-Y., Zhang J.-H., Wei W., Tan Z.-J., Zhang Y., Wu W.-Q., Dai L. (2025). Counterintuitive DNA Destabilization by Monovalent Salt at High Concentrations Due to Overcharging. Nat. Commun..

[64] Endmann A., Baden M., Weisermann E., Kapp K., Schroff M., Kleuss C., Wittig B., Juhls C. (2010). Immune Response Induced by a Linear DNA Vector: Influence of Dose, Formulation and Route of Injection. Vaccine.

[65] Hayashi H., Sun J., Yanagida Y., Otera T., Sasai M., Chang C.Y., Tai J.A., Nishikawa T., Yamashita K., Sakaguchi N. (2022). Modified DNA Vaccine Confers Improved Humoral Immune Response and Effective Virus Protection against SARS-CoV-2 Delta Variant. Sci. Rep..

[66] Boyle J.S., Barr I.G., Lew A.M. (1999). Strategies for Improving Responses to DNA Vaccines. Mol. Med..

[67] Yang H., Zhou Y., Cheng X., Qiu C., Wang S., Xia Y., Huai X., Xiu Z., Wang J., He Y. (2025). Safety, Tolerability, and Immunogenicity of a DNA Vaccine (pGX9501) Against SARS-CoV-2 in Healthy Volunteers: A Single-Center, Randomized, Double-Blind, Placebo-Controlled, and Dose-Ranging Phase I Trial. Vaccines.

[68] Roustazadeh A., Askari M., Heidari M.H., Kowsari M., Askari F., Mehrzad J., Hosseinkhani S., Alipour M., Bardania H. (2025). Enhancing Non-Viral Gene Delivery to Human T Cells through Tuning Nanoparticles Physicochemical Features, Modulation Cellular Physiology, and Refining Transfection Strategies. Biomed. Pharmacother..

[69] Rudolph C., Schillinger U., Ortiz A., Plank C., Golas M.M., Sander B., Stark H., Rosenecker J. (2005). Aerosolized Nanogram Quantities of Plasmid DNA Mediate Highly Efficient Gene Delivery to Mouse Airway Epithelium. Mol. Ther..

[70] Wang S., Zhang C., Zhang L., Li J., Huang Z., Lu S. (2008). The Relative Immunogenicity of DNA Vaccines Delivered by the Intramuscular Needle Injection, Electroporation and Gene Gun Methods. Vaccine.

[71] Rols M.-P. (2006). Electropermeabilization, a Physical Method for the Delivery of Therapeutic Molecules into Cells. Biochim. Biophys. Acta.

[72] Rebersek M., Miklavcic D., Bertacchini C., Sack M. (2014). Cell Membrane Electroporation-Part 3: The Equipment. IEEE Electr. Insul. Mag..

[73] Rols M.P., Teissié J. (1998). Electropermeabilization of Mammalian Cells to Macromolecules: Control by Pulse Duration. Biophys. J..

[74] Kotnik T., Mir L.M., Flisar K., Puc M., Miklavčič D. (2001). Cell Membrane Electropermeabilization by Symmetrical Bipolar Rectangular Pulses: Part I. Increased Efficiency of Permeabilization. Bioelectrochemistry.

[75] Kotnik T., Pucihar G., Rebersek M., Miklavcic D., Mir L.M. (2003). Role of Pulse Shape in Cell Membrane Electropermeabilization. Biochim. Biophys. Acta.

[76] Potter H. (2003). Transfection by Electroporation. Curr. Protoc. Mol. Biol..

[77] Demiryurek Y., Nickaeen M., Zheng M., Yu M., Zahn J.D., Shreiber D.I., Lin H., Shan J.W. (2015). Transport, Resealing, and Re-Poration Dynamics of Two-Pulse Electroporation-Mediated Molecular Delivery. Biochim. Biophys. Acta (BBA)-Biomembr..

[78] Novickij V., Rembiałkowska N., Szlasa W., Kulbacka J. (2022). Does the Shape of the Electric Pulse Matter in Electroporation?. Front. Oncol..

[79] Saulis G., Saulė R. (2012). Size of the Pores Created by an Electric Pulse: Microsecond vs Millisecond Pulses. Biochim. Biophys. Acta.

[80] Satkauskas S., Bureau M.F., Puc M., Mahfoudi A., Scherman D., Miklavcic D., Mir L.M. (2002). Mechanisms of in Vivo DNA Electrotransfer: Respective Contributions of Cell Electropermeabilization and DNA Electrophoresis. Mol. Ther..

[81] Faurie C., Phez E., Golzio M., Vossen C., Lesbordes J.-C., Delteil C., Teissié J., Rols M.-P. (2004). Effect of Electric Field Vectoriality on Electrically Mediated Gene Delivery in Mammalian Cells. Biochim. Biophys. Acta (BBA)-Biomembr..

[82] Li C., Ke Q., Yao C., Yao C., Mi Y., Wu M., Ge L. (2019). Comparison of Bipolar and Unipolar Pulses in Cell Electrofusion: Simulation and Experimental Research. IEEE Trans. Biomed. Eng..

[83] Ibey B.L., Ullery J.C., Pakhomova O.N., Roth C.C., Semenov I., Beier H.T., Tarango M., Xiao S., Schoenbach K.H., Pakhomov A.G. (2014). Bipolar Nanosecond Electric Pulses Are Less Efficient at Electropermeabilization and Killing Cells than Monopolar Pulses. Biochem. Biophys. Res. Commun..

[84] Pakhomov A.G., Grigoryev S., Semenov I., Casciola M., Jiang C., Xiao S. (2018). The Second Phase of Bipolar, Nanosecond-Range Electric Pulses Determines the Electroporation Efficiency. Bioelectrochemistry.

[85] Kotnik T., Miklavcic D., Mir L.M. (2001). Cell Membrane Electropermeabilization by Symmetrical Bipolar Rectangular Pulses: Part II. Reduced Electrolytic Contamination. Bioelectrochemistry.

[86] Lo C.-W., Desjouy C., Chen S.-R., Lee J.-L., Inserra C., Béra J.-C., Chen W.-S. (2014). Stabilizing in Vitro Ultrasound-Mediated Gene Transfection by Regulating Cavitation. Ultrason. Sonochem..

[87] De Cock I., Zagato E., Braeckmans K., Luan Y., de Jong N., De Smedt S.C., Lentacker I. (2015). Ultrasound and Microbubble Mediated Drug Delivery: Acoustic Pressure as Determinant for Uptake via Membrane Pores or Endocytosis. J. Control. Release.

[88] Lentacker I., De Cock I., Deckers R., De Smedt S.C., Moonen C.T.W. (2014). Understanding Ultrasound Induced Sonoporation: Definitions and Underlying Mechanisms. Adv. Drug Deliv. Rev..

[89] Johns L.D. (2002). Nonthermal Effects of Therapeutic Ultrasound: The Frequency Resonance Hypothesis. J. Athl. Train..

[90] Sanwal R., Joshi K., Ditmans M., Tsai S.S.H., Lee W.L. (2021). Ultrasound and Microbubbles for Targeted Drug Delivery to the Lung Endothelium in ARDS: Cellular Mechanisms and Therapeutic Opportunities. Biomedicines.

[91] Yang Y., Li Q., Guo X., Tu J., Zhang D. (2020). Mechanisms Underlying Sonoporation: Interaction between Microbubbles and Cells. Ultrason. Sonochem..

[92] Petkov S., Starodubova E., Latanova A., Kilpeläinen A., Latyshev O., Svirskis S., Wahren B., Chiodi F., Gordeychuk I., Isaguliants M. (2021). Correction: DNA Immunization Site Determines the Level of Gene Expression and the Magnitude, but Not the Type of the Induced Immune Response. PLoS ONE.

[93] Stellwagen E., Stellwagen N.C. (2003). Probing the Electrostatic Shielding of DNA with Capillary Electrophoresis. Biophys. J..

[94] Schriefer L.A., Gebauer B.K., Qui L.Q., Waterston R.H., Wilson R.K. (1990). Low Pressure DNA Shearing: A Method for Random DNA Sequence Analysis. Nucleic Acids Res..

[95] Oefner P. (1996). Efficient Random Subcloning of DNA Sheared in a Recirculating Point- Sink Flow System. Nucleic Acids Res..

